# Light Entrapment by Plasmonic Chiral Lock for Enhancement of 2D Flakes Catalytic Activity

**DOI:** 10.1021/acsami.5c08060

**Published:** 2025-05-21

**Authors:** Anastasiia Tulupova, Denis Zabelin, Andrea Tosovska, Polina Bainova, Mariia Erzina, Anna Zabelina, Vasilii Burtsev, Anastasiia Skvortsova, Marie Urbanova, Martin Kartau, Affar S. Karimullah, Vaclav Svorcik, Oleksiy Lyutakov

**Affiliations:** † Department of Solid State Engineering, University of Chemistry and Technology, 16628 Prague, Czech Republic; ‡ Department of Physics and Measurements, University of Chemistry and Technology, 16628 Prague, Czech Republic; § School of Chemistry, 3526 University of Glasgow, Joseph Black Building, Glasgow G12 8QQ, U.K.

**Keywords:** chiral gold helicoids, MoS_2_ flakes, plasmon coupling, plasmon-assisted chemistry, nitrogen reduction

## Abstract

Plasmon-based triggering leads to an effective increase of material catalytic activity in a number of relevant photoelectrochemical transformations, including nitrogen reduction for the production of ammonia. The efficiency of the plasmon assistance can be significantly increased through the rational design of hybrid photoelectrodes, e.g., by placing a redox-active material at plasmonic hot spots that may arise between two coupled nanostructures. In this work, we describe the creation and utilization of chiral plasmon-active hybrid structures (based on the so-called gold helicoids) coupled with redox-active 2H-MoS_2_. The chiral plasmon-active gold nanoparticles (with the same or opposite chirality) were spatially separated by thin two-dimensional (2D) flakes to reach mutual plasmon coupling between them. Using numerical simulations and SERS measurements, the dependence of the local enhancement of the electric field (EF) inside the created plasmon-active diastereomer consisting of Au helicoid–2D MoS_2_–Au helicoid “sandwich structure”, on the mutual chirality of the nanoparticles is demonstrated. It is found that the plasmon energy is more efficiently “concentrated” in the MoS_2_ space using the “chiral trap” of light energy (i.e., chiral plasmonic lock), even in the case where the chiral handedness of Au nanoparticles is matching. The created hybrid structures were subsequently used for nitrogen reduction and ammonia production proceeding on the MoS_2_ surface. A clear dependence of the catalytic activity of MoS_2_ on the matching or mismatching of Au helicoid chiralities (and related local value of EF) is observed. In particular, a two-time increase in the ammonia yield is obtained in the case of matching chirality, compared to that in the case of mismatched configuration or the control experiments performed with nonchiral Au nanocubes. Hence, the utilization of chiral plasmonic nanoparticles and their dimers (or multimers) provides an additional opportunity for even more effective photosensibilization of redox-active materials.

## Graphical abstract

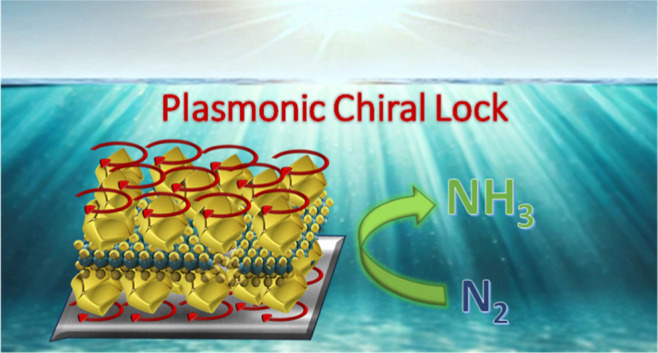

## Introduction

Plasmon, as a collective oscillation of free electrons in metal nanostructures, can ensure the efficient coupling of incident (i.e., focusing) photon energy in the nanospace close to a metal nanostructure.^1^ This phenomenon finds a wide application in the field of sensors, optoelectronics, and photo- or photoelectrocatalysis.^2–4^ In the last case, the plasmon active nanoparticles are combined with a redox-active material, ensuring the efficient light absorption and triggering catalyst activity.^5–7^ As plasmon-active structures, involved in the design of such hybrid photoelectrodes, various nanostructures with different metals, commonly silver, copper, or gold, more rarely UV-light absorbing ones, like aluminum, are used.^8,9^ Among them, Au nanostructures deserve special attention due to their chemical stability and strong absorption of a significant part of the sunlight energy.^10^ Based on the reaction type, the plasmon-active Au nanoparticles can be combined with different redox-active materials, such as metal sulfides, metal oxides, nitrides, and metal free catalysts as well as various ternary compounds.^11–17^ In turn, such Au nanoparticles, which are spherical, in the form of nanorods, gold nanourchins or nanostars, and clusters as well in other various shapes (sometimes undefined), have been reported.^11–13,15^ Most of the related research has been focused on water splitting,^11–14,18^ but recently, the attention has been turned to such energy-demanding and relevant reaction as nitrogen reduction.^19–22^

Various mechanisms are considered for plasmon-assisted enhancement of the redox activity of plasmon-triggered materials, including the plasmon-related near field-induced electron–hole pair generation and separation, injection of hot electrons of holes in the semiconductors, and plasmon-induced local heating.^11–15,23^ In all cases, related mechanisms and phenomena are determined by larger plasmon-related near-field enhancement. Careful design of plasmon-active nanostructures will ensure higher near field enhancement, increasing the efficiency of plasmon-assisted catalysis in this way.^23,24^ To enhance the local electric field (EF), several strategies were proposed, including the optimization of Au nanostructures’ shape and size, creation of Fabry–Perot resonators, or utilization of plasmon coupling.^25–27^ Even plasmon coupling can provide a very high field enhancement, leading to strong confinement of plasmonic energy in the hot spot between two closed nanostructures.^28–33^ In this regard, the efficiency of plasmon triggering is a function of the coupled plasmon-active nanostructures and space between them.

Recently, the new kind of plasmon active nanoparticles—chiral nanoparticles or so-called helicoids—were reported.^34–37^ In combination with subwavelength confinement of electromagnetic fields, the gold helicoids can support the excitation of chiral electromagnetic near field restricted to a few nanometres.^38–40^ In our recent paper,^41^ we demonstrated that the strength of the plasmon coupling between two chiral nanostructures is a function of their chirality. Specifically, the coincidence of chirality results in a higher enhancement of the local electromagnetic field (excited under illumination with unpolarized light). Since the mechanism of plasmon-related catalysis is also determined by the local value of EF,^42,43^ it can be expected that the interplay of chirality in the case of coupled gold helicoids will also affect the redox properties of the material, sandwiched in the space of chiral coupled plasmon. In other words, the chirality in the utilization of Au nanostructures for catalysis may open an additional degree of freedom for the additional enhancement of plasmon coupling and more efficient redox activity triggering of the closed material.

Based on this idea, we propose the utilization of chirality-dependent plasmon coupling (a structure that can be called a “chiral plasmonic lock”) between gold helicoids for the triggering of the catalytic activity of sandwiched two-dimensional (2D) MoS_2_ flakes. The advantages of the created chiral photoelectrodes were subsequently verified in nitrogen reduction reaction.

## Results and Discussion

### General Concept and Experimental Route Used for Chiral Plasmonic Lock Creation

The main concept of our work is depicted in Figure 1, which includes sample preparation, utilization, and potential dependency of plasmon triggering of MoS_2_ redox activity on the coincidence or mismatch of two introduced chirality centers. As was mentioned above, the sandwiching of redox-active MoS_2_ flakes between chiral plasmon active nanoparticles was realized (Figure 1A) by using step-by-step deposition of Au helicoids and 2D flakes.

**Figure 1:**
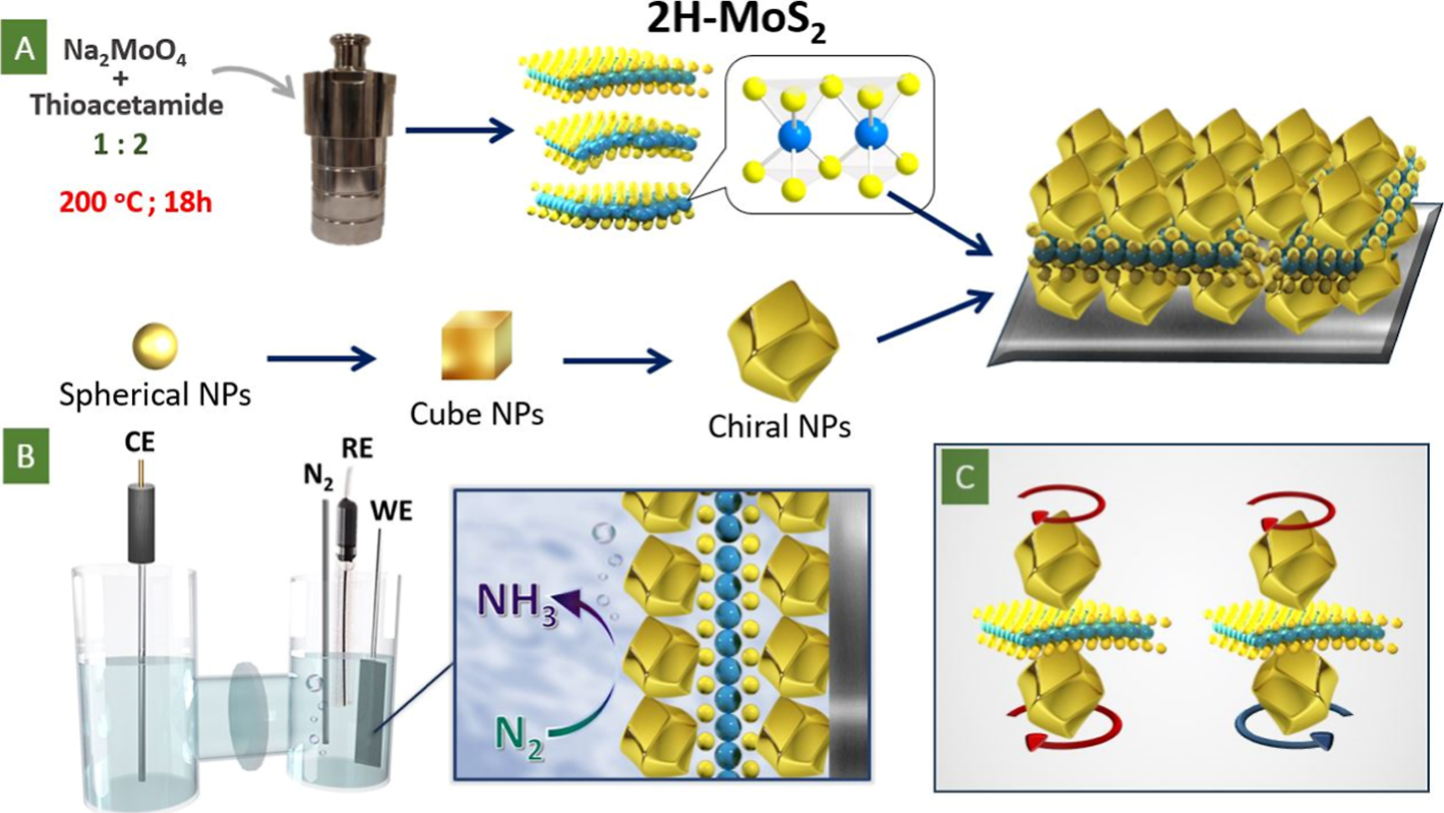
(A) Schematic representation of samples preparation, aimed at the sandwiching of 2D MoS_2_ flakes between chiral Au nanoparticles with the same or opposite chirality; (B) utilization of created structures in NRR using the photoelectrochemical route; and (C) proposed dependency of coupled plasmon triggering on the Au helicoids’ chirality coincidence or mismatch.

First, the monolayer of Au helicoids, created from cubic nanoparticles (Figures S2–S5) with utilization of amino acid partial surface blockage, was deposited on the titanium substrate by drop deposition and slow solvent evaporation. During the preparation of chiral Au helicoids from Au cubes, there is an increase in the nanoparticle size (Figures S6 and S7), leading to a shift of the plasmon absorption band position from 544 to 577 nm (Figure S8). Subsequently, MoS_2_ flakes, prepared by the solvothermal approach (Figures S9 and S10), were added on the top of the closely packed layer of Au helicoids. Deposition of MoS_2_ was optimized to achieve a surface closed to the ideal monolayer of flakes covering the surface (see Supporting Information for details). In the final step, the Au helicoids with the same or opposite chirality were deposited on the surface of MoS_2_ flakes, creating the proposed sandwiched system.

The catalytic activity of the sandwiched flakes was subsequently triggered by a strong plasmonic field under the sample surface illumination at an angle of 30–45° to the surface normal (noncollimated light was used). This leads to modes being generated that generate strong electromagnetic field confinement between the top and bottom layers of AuNPs. The efficient plasmonic coupling was ensured by the 2D nature of MoS_2_, which provides a nanometer-scale gap between the top and bottom layers of the Au helicoids. The created structures were subsequently used for nitrogen reduction, performed in the photoelectrochemical mode (Figure 1B). The excited plasmonic field is chiral, in the sense that the coincidence (or mismatch) of the chirality of the local plasmonic fields will be responsible for stronger (or weaker) excitation of chiral plasmonic hot spots and corresponding activation of MoS_2_ flakes (Figure 1C). In other words, the proposed system using plasmonic diastereomers enables controllable catalytic activity from the 2D material.

### Characterization of the Particular Materials Used

Characterization of the particular materials used is presented in Figure 2. First, the results of X-ray diffraction (XRD measurements (Figure 2A) of MoS_2_ flakes reveal the appearance of several characteristic reflexes that correspond well with the previously reported pattern (JCPDF 37-1492). The presence of low angle reflex confirms the 2D nature of the flakes. Raman spectroscopy shows the appearance of two characteristic peaks, located at 382 cm^–1^ and 407 cm^–1^, attributed to in-plane mode E2g1 and out-of-plane mode A_1g_ vibrations in the flakes structure (Figures 2B and S10). The 2D nature of MoS_2_ flakes was definitively confirmed by atomic force microscopy (AFM) measurements, depositing flakes on the Ti substrate from a diluted suspension (Figure 2C vs Figure S9). The flake thickness of about 5 nm provides a suitable distance for efficient coupling of chiral plasmon active NPs.

**Figure 2:**
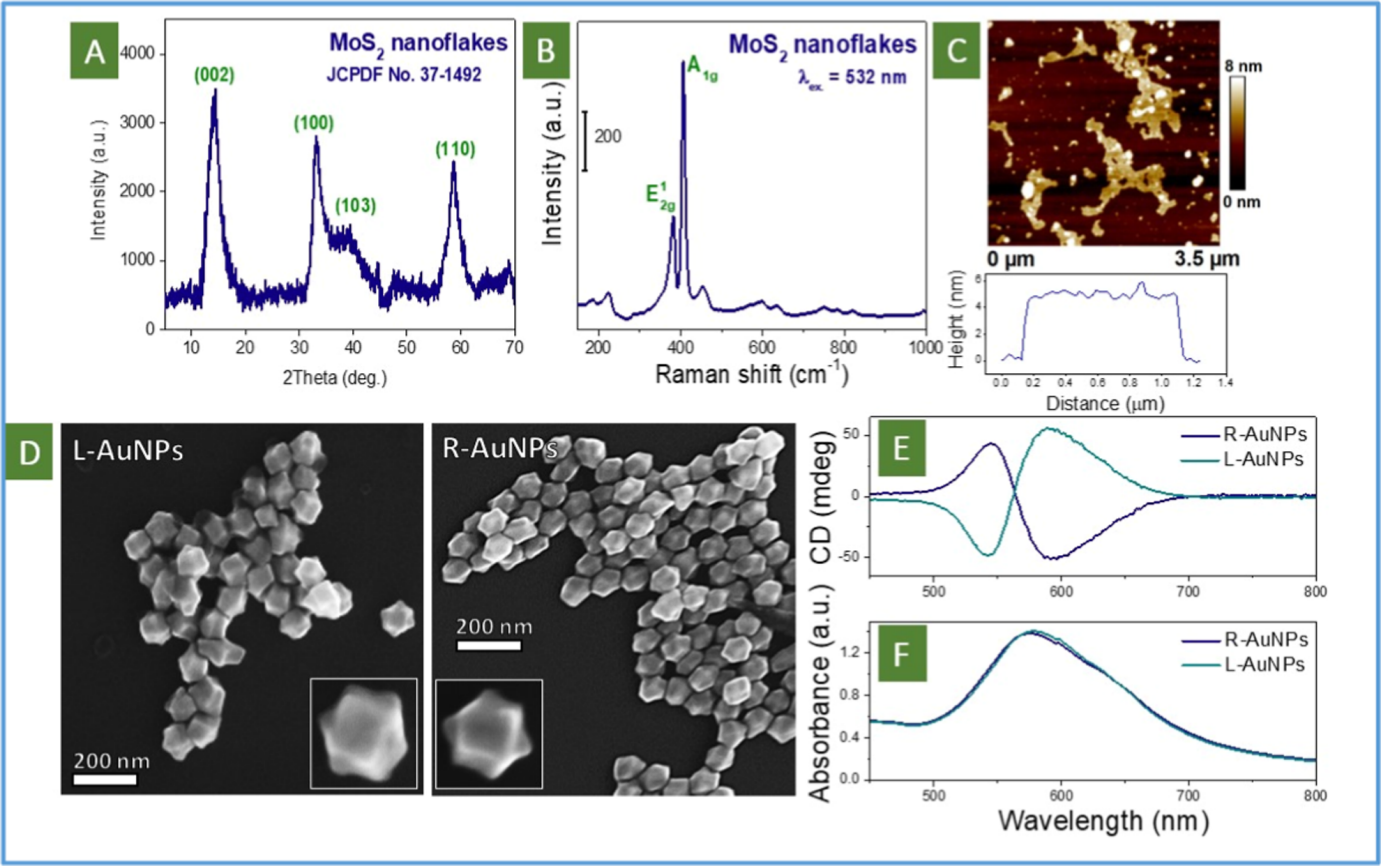
Characterization of separated elements’ structure: (A) XRD pattern, (B) Raman spectrum, and (C) AFM-measured morphology of MoS_2_ flakes; (D) SEM image of left- and right-handed Au helicoids; (E) CD and (F) UV–vis spectra of left- or right- handed Au helicoids.

The AuNPs show a specific geometry, recently reported for this kind of plasmon-active and chiral nanoparticles (Figure 2D shows the surface geometry of Au helicoids after several times of washing).^36,49^ Strong washing allows one to estimate the “real” surface morphology of Au helicoids but can prevent the nanoparticles from self-assembling. Thus, for subsequent formation of the closely packed Au helicoids layer, less careful washing has to be used. In addition, the curved geometry of the nanoparticle edges is also evident from transmission electron microscopy (TEM) measurements (Figure S11—see gray regions corresponding to thinner gold thickness on curved nanoparticles edges and more transparent to the electron beam than the nanoparticle “core”).

Measured UV–vis spectra of the suspension of Au helicoids show a broad plasmonic absorption band located at 500–700 nm (Figure 2F). At these wavelengths, the appearance of well-visible circular dichroism (CD) signals due to differential absorption of left- or right-handed circularly polarized light was also observed (Figure 2E). As previously reported, such optical chirality arises due to a specific shape of the created Au nanoparticles (Figure 2D). The calculated value of the *g*-factor at 590 nm, as a quantitative chirality parameter, was found to be 0.0024, significantly outperforming most “natural” objects (such as small or large organic chiral biomolecules). It should also be noted that we used the route proposed by Nam’s group, which produced several types of intrinsically chiral nanoparticles. Some of them, such as 432 helicoid III, have greater chirality,^36^ but do not show a tendency to self-assemble and to form an ordered array of closely packed helicoids, which is crucial for the application reported here. Hence, we used helicoids type IV,^49^ which have a lower value of *g*-factor, but can self-assemble easily on a flat substrate.^50^

### Characterization of the Chiral Plasmonic Lock Structure

The evolution of sample surface morphologies during the particular material depositions is presented in Figure 3A–C (optimization of samples preparation is described in Supporting Information—Figure S12 and related discussions). Briefly, the main efforts were devoted to reaching a structure, where the monoflake layer of MoS_2_ will be sandwiched between monolayers of Au helicoids. The first Au helicoid deposition and the MoS_2_ layer depositions were relatively straightforward and easily produced good results—Figure 3A shows the closely packed array of helicoids, while Figure 3B reveals the homogeneous coating of helicoids by MoS_2_ flakes. The final deposition of helicoids does not produce such a perfect coating as in the previous cases (Figure 3A vs Figure 3C). However, the created coating can fulfill both tasks, to ensure plasmon coupling from one side and to leave part of the MoS_2_ surface uncovered, i.e., able to be in contact with the electrolyte. Similar results were observed using AFM characterization of surface morphology. In this case, the appearance of additional surface features can be attributed to the Au helicoid deposition (Figure S13). In the last step, the gap between Au helicoids was estimated using scanning electron microscopy (SEM) cross-section measurements as well as AFM scratch tests and found to be below 10 nm (Figures S13 and S14).

**Figure 3:**
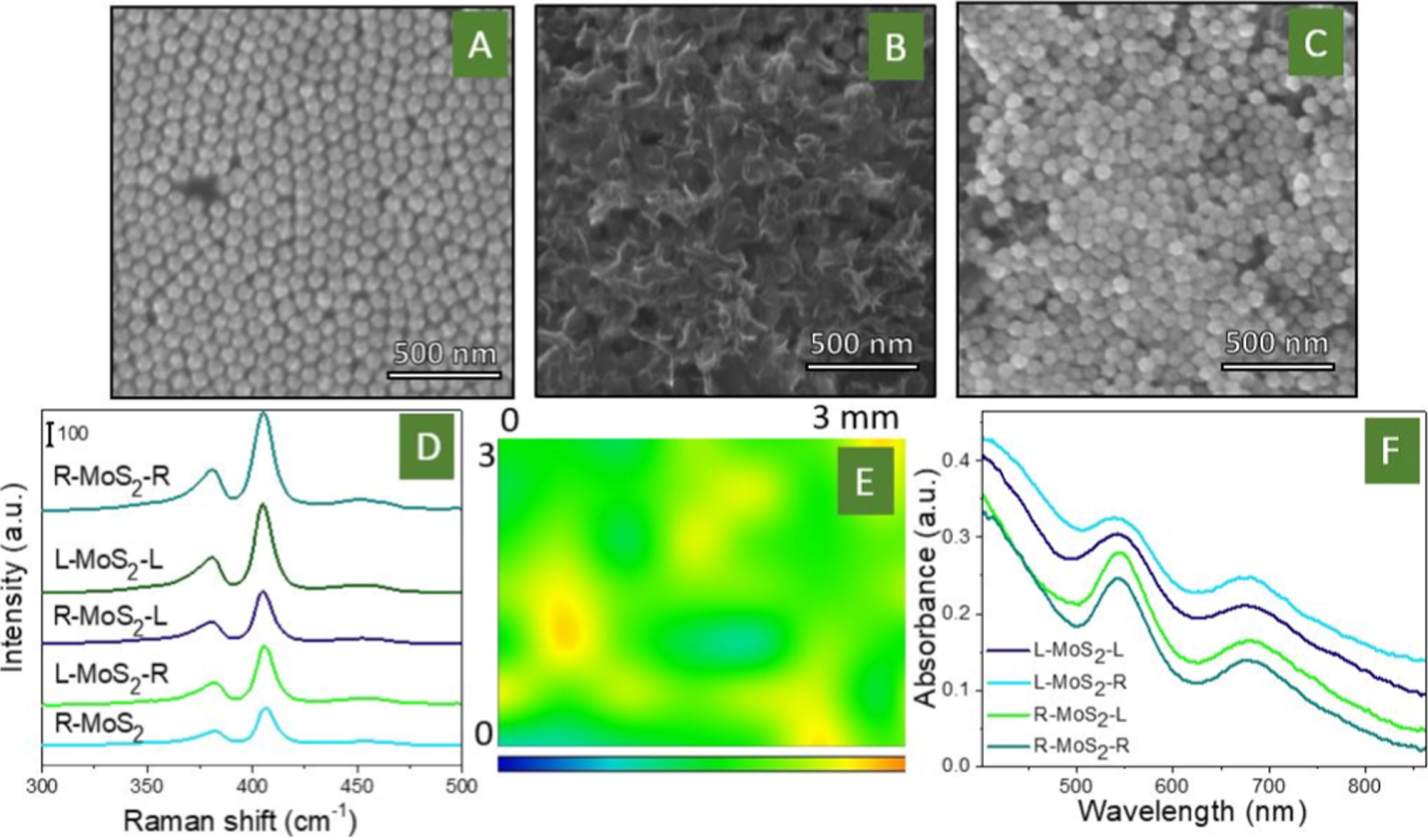
(A–C) SEM images of Au helicoid array deposited on the conductive electrode surface, Au helicoids covered by MoS_2_ flakes, and the final sandwiched structure (R-MoS_2_-R) surface morphology; (D) SERS spectra of MoS_2_ flakes deposited on the Au helicoid surface or sandwiched between Au helicoids; (E) map of MoS_2_ characteristic Raman peak intensity, measured across 3 × 3 mm^2^ area; and (F) UV–vis absorption spectra of the sandwiched structures measured in the reflection mode.

In turn, the SERS spectra of MoS_2_ flakes, deposited on the Au helicoid surface or sandwiched between helicoids, are presented in Figures 3D and S15. The spectra were averaged from the SERS mapping results to avoid errors due to random deviations in the local arrangement of nanoparticles/flakes. Results prove that the sandwich-like structure with MoS_2_ flakes between nanoparticles leads to an increase in the intensity of characteristic MoS_2_ SERS peaks. Moreover, the intensity of SERS peaks was found to be a function of nanoparticle combination: for different chiralities (R–L), it was smaller than the matching chirality (R–R) combination. Since the SERS intensity is directly related to the local value of the plasmon-related EF, i.e., the local strength of the plasmon, we can conclude that the chirality of Au helicoids really plays a significant role in plasmon coupling. In particular, a higher local value of the plasmon energy is achieved for R–R nanoparticles, also leading to a more pronounced enhancement of the MoS_2_ SERS response. Figure 3E shows the SERS distribution of the characteristic MoS_2_ peak, located at 407 cm^–1^ for the R-MoS_2_-R case. The homogeneous distribution of peak intensity confirms the uniform covering of the sample surface by flakes and the relatively similar strength of plasmon coupling across the macroscopic surface area. Generally, SERS measurements allow us to draw a few important conclusions: (i) the plasmon coupling actually occurs (evident from the comparison of MoS_2_ flakes only placed on Au and those sandwiched between two Au nanoparticles); (ii) the strength of plasmon coupling is a function of the nanoparticles mutual chirality; and (iii) plasmon coupling is homogeneous across the sample surface.

Finally, the UV–vis spectrum of the proposed sandwiched structure, measured in reflection mode under underwater conditions, is presented in Figure 3F. Interestingly, a splitting of the plasmon absorption bands is observed after the sample is immersed in water (however, this result correlates well with the numerical simulation described below). Unfortunately, it was not possible to measure the samples using chiral light in the reflected mode underwater, so we performed measurements in the transmission mode (Figure S16). However, in this case, the transmitted light interacted with a maximum of two monolayers of nanoparticles, so the obtained CD signal was very weak. In addition, chirality in the far field (i.e., observed using CD measurements) and near field (chiral distribution of plasmon-related closed field) may not correlate.

We also compared the CD spectra of different combinations of right- or left-handed Au helicoids, separated by the MoS_2_ layer. In this case, the appearance of a chiral response was observed in the case of matched chirality, and it is almost complete absence in the case of incorrect chirality. However, this result cannot reveal the local value of the EF (attributed to the plasmonic near field), since CD measurements are made in the “far field” and represent the averaged value of the response of the entire system. Therefore, we used an alternative approach described in the following to estimate the local value of the plasmon-related EF in the case of the coinciding or mismatched chirality of the nanoparticles.

## Estimation of Plasmon Coupling for Matched and Mismatched Au Helicoid Combination with Utilization of the PS Layer

For the estimation of near-field plasmon enhancement as a function of matching or mismatching of Au helicoid chirality, additional experiments with utilization of the sandwiched polystyrene (PS, with a thickness below 10 nm) layer were performed. A schematic representation of the used experimental concept is given in Figure 4A. The enhancement of the SERS signal from PS was expected to reveal the relative value of the local EF (since the absorption of PS is far from the used excitation wavelengths or the AuNP plasmon absorption band and such mechanisms of Raman signal enhancement as resonance Raman or chemical enhancement can be excluded). The results obtained are presented in Figure 4C–F, and as is evident, the response of PS is a real function of nanoparticle chirality matching or mismatching. The following bands were observed: 620 cm^–1^ (ring deformation mode), 796 cm^–1^ (C–H out of plane), 1000 cm^–1^ (ring breathing mode), 1031 cm^–1^ (C–H in plane deformation), 1154 cm^–1^ (C–C stretch), 1448 cm^–1^ (CH_2_ scissoring), 1580 cm^–1^ (C=C stretch), and 1600 cm^–1^ (ring skeletal stretch). The more pronounced band (1000 cm^–1^) was subsequently used for estimation of the SERS enhancement.

**Figure 4:**
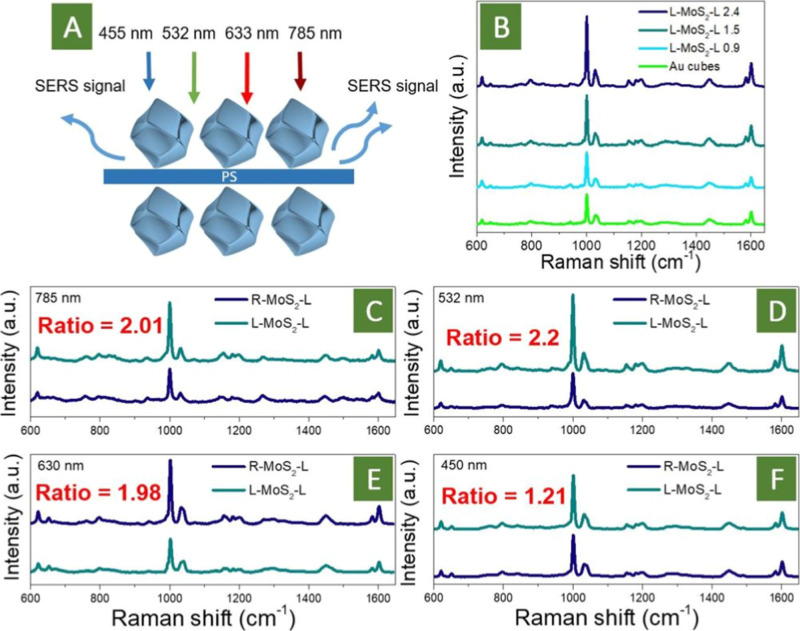
(A) Schematic representation of a local value of plasmon-related field estimation, for the PS layer sandwiched between Au nanoparticles with different chiralities; (B) SERS spectra of the PS layer, sandwiched between less chiral L-AuNPs (chirality is given as *g* factor * 10^–3^) and cubic AuNP (532 nm excitation wavelength); (C–F) SERS spectra as a function of chirality of the nanoparticles matched or mismatched for different excitation wavelengths (SERS enhancement is given as a ratio, calculated for the 1000 cm^–1^ band).

With utilization of 532 nm excitation wavelength, the more pronounced Raman bands were observed for the L–L AuNP case, indicating that even in this case, the higher value of the local EF (plasmon-related near field) is reached. On the contrary, the utilization of nanoparticles with opposite chirality led to a less pronounced PS response. A similar situation was also observed at 633 and 785 nm excitation wavelengths (which is in good agreement with UV–vis spectra obtained in the reflected mode). However, for the 455 nm excitation wavelength, almost no difference was observed, which is expected because this wavelength is far from the optical range, where the chirality of Au helicoids was observed (Figure 3F).

So, we demonstrated that the local value of the EF excited in the space between the Au helicoid layers is really a function of the nanoparticles chirality match or mismatch. This phenomenon was observed solely for wavelengths that correspond to the appearance of the chiral plasmon. A similar phenomenon can be expected (and demonstrated, see Figure 3D) for the MoS_2_ case. Since the plasmon triggering of MoS_2_ is carried out even by the plasmon-related field (or accompanied phenomena, such as hot electrons injection, which is also a function of the local EF), it can be expected that the redox activity of the sandwiched MoS_2_ layer will also be determined by the Au nanoparticles’ matched or mismatched chirality, which was studied in the subsequent experiments.

We also performed a range of experiments with the PS layer sandwiched between helicoids with a lower value of chirality. For this goal, helicoid synthesis was stopped after 15 and 30 min (nanoparticles shape is presented in Figure S17), leading to a less pronounced CD signal (spectra are presented in Figure S18), according to the experimental observation reported in ref 37. In contrast, further increase in the time required to synthesize Au helicoids, compared to the commonly used 60 min, does not lead to a chirality increase (see Figure S19). We used the 532 nm excitation wavelength, since more apparent dependence on the Au helicoid matching or mismatching is expected at this wavelength (Figure 4B). As is evident, when the chirality of nanoparticles decreases, the SERS intensity of PS response also drops, up to the value that is characteristic of control, nonchiral case of Au cubes. So, this result additionally highlights the apparent contribution of the chirality matching to local plasmon coupling and plasmonic near-field enhancement.

### Nitrogen Reduction in Chiral Plasmonic Lock

In the next step, the sandwiched structure created was used for the NRR (proceeding on the surface of MoS_2_), performed in the photoelectrochemical mode. Samples were illuminated under varied angles, relative to the surface normal, to excite the plasmonic dipole(s), consisting of upper and lower Au helicoids, and to ensure efficient plasmonic activation of the sandwiched MoS_2_ layer (Figure S20). First, the linear sweep voltammetry (LSV) curves were measured for the R–R and R–L combination of nanoparticles in the dark and under the light illumination. We used the N_2_-saturated electrolyte, and concurrent to NRR, the hydrogen evolution was blocked utilizing the addition of an ionic liquid (20 wt % ([C4mpyr]­[eFAP])). The shift of LSV curves toward the lower potential, observed under the sample illumination, indicated that the NRR reaction proceeds more efficiently under plasmon triggering (Figure 5A). In both cases of the R–R and R–L helicoid combinations, the redox activity of sandwiched MoS_2_ flakes was enhanced by plasmon triggering (due to a local enhancement of plasmon-related EF, which triggers the redox activity). However, for the R–R combination of helicoids (for chirality coincidence), the shift of the LSV curves was more pronounced than for the R–L combination, revealing the greater impact of plasmon triggering (i.e., higher local value of EF). In addition, electrochemical impedance spectroscopy (EIS) measurement results (Figure S21) also indicate the significant impact of plasmon triggering and the interplay of nanoparticles chirality. In particular, the highest apparent decrease of the charge transfer resistance (between MoS_2_ and electrolyte) was observed for all cases of plasmon triggering (evident as an apparent decrease of semicircle radii on EIS spectra). However, in the case of the coincidence of nanoparticle chirality, more significant decrease of charge transfer resistance was observed, compared to that in the case of Au helicoids with mismatched chirality. These results correspond well to previous SERS measurements and additionally highlight the role of nanoparticle chirality for plasmonic coupling.

**Figure 5:**
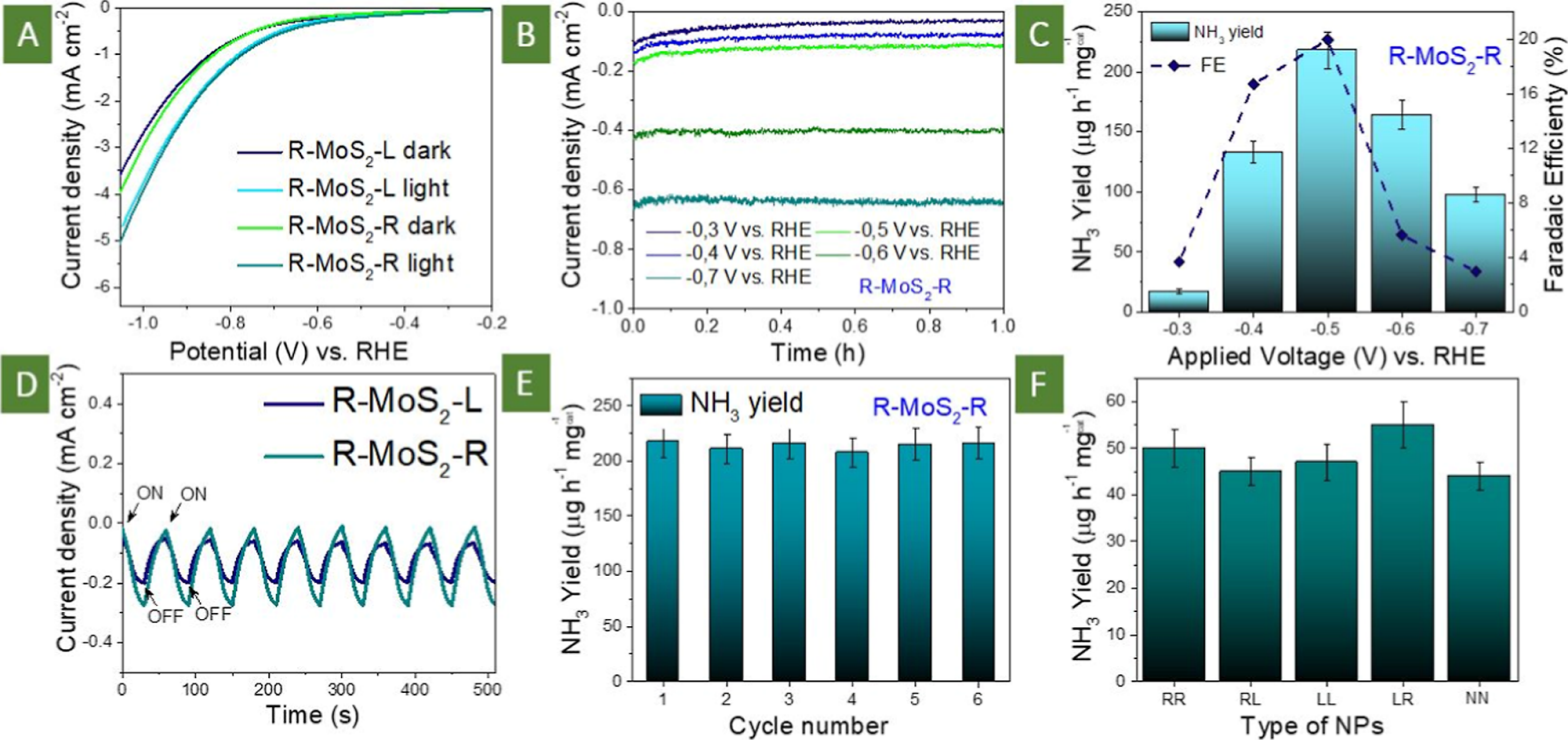
(A) LSV curves measured with utilization of R-MoS_2_-R or R-MoS_2_-L photoelectrodes in the dark or under illumination; (B) chronoamperometry curves, obtained with utilization of R-MoS_2_-R photoelectrode and different potentials; (C) NH_3_ yield and Faradaic efficiency obtained within photoelectrochemical mode with utilization of the R-MoS_2_-R photoelectrode; (D) several subsequent cycles of light switching ON/OFF demonstrating the fast response of R-MoS_2_-R and R-MoS_2_-L photoelectrode; (E) results of stability tests with each cycle duration of 1 h; (F) NH_3_ production yield reached in the dark with utilization of MoS_2_ sandwiched between Au nanoparticle layers (NN case corresponds to nonchiral Au nanocubes).

In the next step, the chronoamperometry measurements were performed at constant potentials (Figure 5B) and the amount of produced ammonia was estimated as a function of the applied potential (Figure 5C) using separately prepared calibration curves (Figure S22). In this case, we used R-MoS_2_-R samples since better NRR results could be expected for them. The observed dependencies of both the NH_3_ yield and the Faradaic efficiency have an optimal value of potential, which is 0.5 V in our case vs reversible hydrogen electrode (RHE). In addition, similar results but with apparently less efficiently were obtained with the utilization of mismatched chirality combination (Figure S23), since in this case, the lower local value of plasmon-related EF was reached. All further experiments with RHE were performed using this potential. The experiments with simple MoS_2_ flakes (without plasmon triggering) or MoS_2_ flakes deposited on a single layer of helicoids or Au nanocubes (that is, without plasmon coupling) result in significantly lower NH_3_ production (see the results of control experiments, presented in Figuress S24 and 5F: 53.51 μg h^–1^ mgcat^–1^ for MoS_2_ flakes and 73.34 μg h^–1^ mgcat^–1^ for Au nanocube-MoS_2_ structure and compare them with 217.93 μg h^–1^ mgcat^–1^ reached with the utilization of R-MoS_2_-R photoelectrode). A similar decrease in catalyst efficiency was observed without sample illumination (Figure 5F) (notably, no differences were observed for the “dark” experiments with the use of R–R, L–L, R–L, L–R or coupled nonchiral Au nanocube systems (Figure S25). Even more, an apparent decrease of created structure redox activity was observed with utilization of the wavelength(s), not overlapping with the plasmon absorption band (Figure S26). As expected, NH_3_ was produced solely on the MoS_2_ surface; removing MoS_2_ from the catalytic system results in a close to zero ammonia yield. It should also be noted that the amount of “undesired” N_2_H_4_, created with the R-MoS_2_-R photoelectrode utilization was negligible (Figure S27). The impact of coupled plasmon triggering was also highlighted by the light switching ON/OFF experiments, performed with the utilization of R–R nanoparticle combination (Figure 5D). Light illumination immediately leads to an increase in current density due to the plasmon-assisted triggering of MoS_2_ redox activity.

Such a rapid increase, within a few seconds, can be attributed to electronic processes inside the flakes and not to any plasmonic heating effects (which commonly take a few minutes in the steady-state illumination regime with medium power on the surface).^51^ In addition, power-dependent experiments also indicate the linear dependency between irradiance and ammonia yield (Figure S28), which also indicate direct photocatalysis, without involving an intermediate “heating” stage. Finally, a simple heating of the system (60 °C) results in the reaction efficiency decrease, probably due to the decreased solubility of nitrogen in the reaction mixture (Figure S29). In addition, several subsequent cycles of NH_3_ production indicate that the catalytic activity of the created sandwiched photoelectrode is stable; no decrease in the amount of NH_3_ produced was observed (Figure 5E, the corresponding current density is given in Figure S30). In turn, conservation of the structure stability was also observed for the case of mismatched chirality (Figure S31). Finally, Raman and SEM measurements performed after the stability tests (R-MoS_2_-R samples) also indicate the conservation of the morphology and structure of the samples (Figures S32 and S33).

So, from the experiments described above, we can conclude that the plasmon coupling, reached with the chiral gold nanoparticles, can increase the redox activity of sandwiched MoS_2_ flakes. Moreover, the efficiency of plasmon coupling is closely related to the mutual nanoparticles chirality interplay and reached local EF value in the space of plasmonic hot spot. The most efficient production of NRR was reached with the utilization of −0.5 vs RHE external bias and nanoparticles with the same chirality. Even under these conditions, the value of Faradaic efficiency reaches 19.94%, which is very good for the NRR result (Table 1),^52–65^ while the overall stability of the used R-MoS_2_-R photoelectrode is relatively good. Comparison with the data from the current literature (Table 1) shows that the FA value obtained in this work is lower than those in some published studies. On the other hand, we did not use increased N_2_ pressure or temperature in the reaction chamber (experiments were performed at RT and atmospheric pressure). Even in this case, we were able to achieve a high yield of NH_3_. In addition, in this work, we focus more on the fundamental possibility of using a chiral plasmonic dipole to increase the redox activity of the sandwiched material. In the future, it will be possible to increase FA using alternative redox-active materials that could suppress hydrogen evolution, addition of ionic liquid, or incorporation of single atom redox active centers.

**Table 1:** Comparison of the Present Results with Previously Published Ones

catalyst	electrolyte	applied potential (vs RHE) (V)	NH_3_ yield	FE (%)	refs
Re-In_2_Se_3_-VIn/CC	0.1 M Na_2_SO_4_	–0.5	26.63 μg h^–1^ cm^–2^	30.8%	^52^
MoN-NV_2_	0.1 M KOH	–0.3	22.5 μg h^–1^ mg_cat_^–1^	14%	^53^
Fe_2_(MoO_4_)_3_/C	0.1 M Na_2_SO_4_	–0.4	51.76 μg h^–1^ mg_cat_^–1^	90.63% at –0.35 VRHE	^54^
MoSe_2_/Ti_3_C_2_	0.05 M H_2_SO_4_	–0.55	60.87 μg h^–1^ mg_cat_^–1^	9.3% at –0.25 V	^55^
WSeS/WSe_2_	0.1 M Na_2_SO_4_	–0.3	15.96 μg h^–1^ mg_cat_^–1^	40.2%	^56^
MoO3–*x*/MXene	0.5 M LiClO_4_	–0.4	95.8 μg h^–1^ mg_cat_^–1^	22.3% (−0.3 V)	^57^
PdFe single-atom alloy metallene	0.5 M LiClO_4_	–0.2	111.9 μg h^–1^ mg_cat_^–1^	37.8%	^58^
WSe2–*x*	12 M LiClO4	–0.5	181.3 μg h^–1^ mg_cat_^–1^	62.5%	^59^
SV-rich heterostructured 1*T*/2H-MoS_*x*_ monolayer	0.1 M Na_2_SO_4_	–0.4	93.2 μg h^–1^ mg_cat_^–1^	20.5%	^60^
Co–MoS_2_/N@C	0.1 M Na_2_SO_4_	–0.4	129.93 μg h^–1^ mg_cat_^–1^	11.21%	^61^
Ni–Fe@MoS_2_ NCs	0.1 M Na_2_SO_4_	–0.3	128.17 μg h^–1^ mg_cat_^–1^	11.34%	^62^
TiO_2_–x@COF-Aq	0.1 M HCl	–0.5	30 μg h^–1^ mg_cat_^–1^	16%	^63^
MoSSe	0.1 M HCl	–0.45	32.32 μg h^–1^ mg_cat_^–1^	12.66%	^64^
Cu-doped Fe_2_O_3_ NRs	0.1 M Li2SO4	–0.15	12.5 μg h^–1^ mg_cat_^–1^	16.4%	^65^
R-MoS_2_-R	0.1 M Na_2_SO_4_	–0.5	217.93 μg h^–1^ mg_cat_^–1^	19.94%	our work

The catalytic activity of MoS_2_ as a function of nanoparticle chirality is illustrated in Figure 6A (the amount of NH_3_ produced and the Faradaic efficiency are used as a marker). Measurements were carried out with the use of various combinations of nanoparticles, including L–L, R–R, L–R, and R–L ones, as well as the utilization of nonchiral cubic AuNPs (designated as N–N, where “N” represents nonchiral Au nanoparticle). As is evident, the amounts of NH_3_ produced with the use of L–R and R–L are similar. Moreover, equal amounts were reached with the implementation of MoS_2_ sandwiched between nonchiral cubic AuNPs (N–N case). However, in the case of chirality coincidence, ca. 2 times increase in NH_3_ yield was observed and can be attributed to a higher local value of EF. A similar situation was observed for the Faradaic efficiency, where the apparent contribution from matching chirality also led to a significant increase of its value. Hence, the contributions of matching chirality to the plasmonic coupling are proven to have a significant impact on the triggering of the catalytic activity of MoS_2_ (compared to both mismatched chirality and nonchiral nanoparticles cases) and therefore the utilization of plasmon-active diastereomers makes sense. It should also be noted that in the case of nonchiral nanoparticles, the intersection of light-emitting diode (LED) emission wavelengths and plasmon absorption band was similar to that in the case of chiral nanoparticles (difference was less than 20%; a more detailed description is given in Supporting Information—Figure S34 and related discussion), so the matched chiral plasmon triggering bring additional enhancement of local field and related increase of sandwiched material catalytic activity.

**Figure 6:**
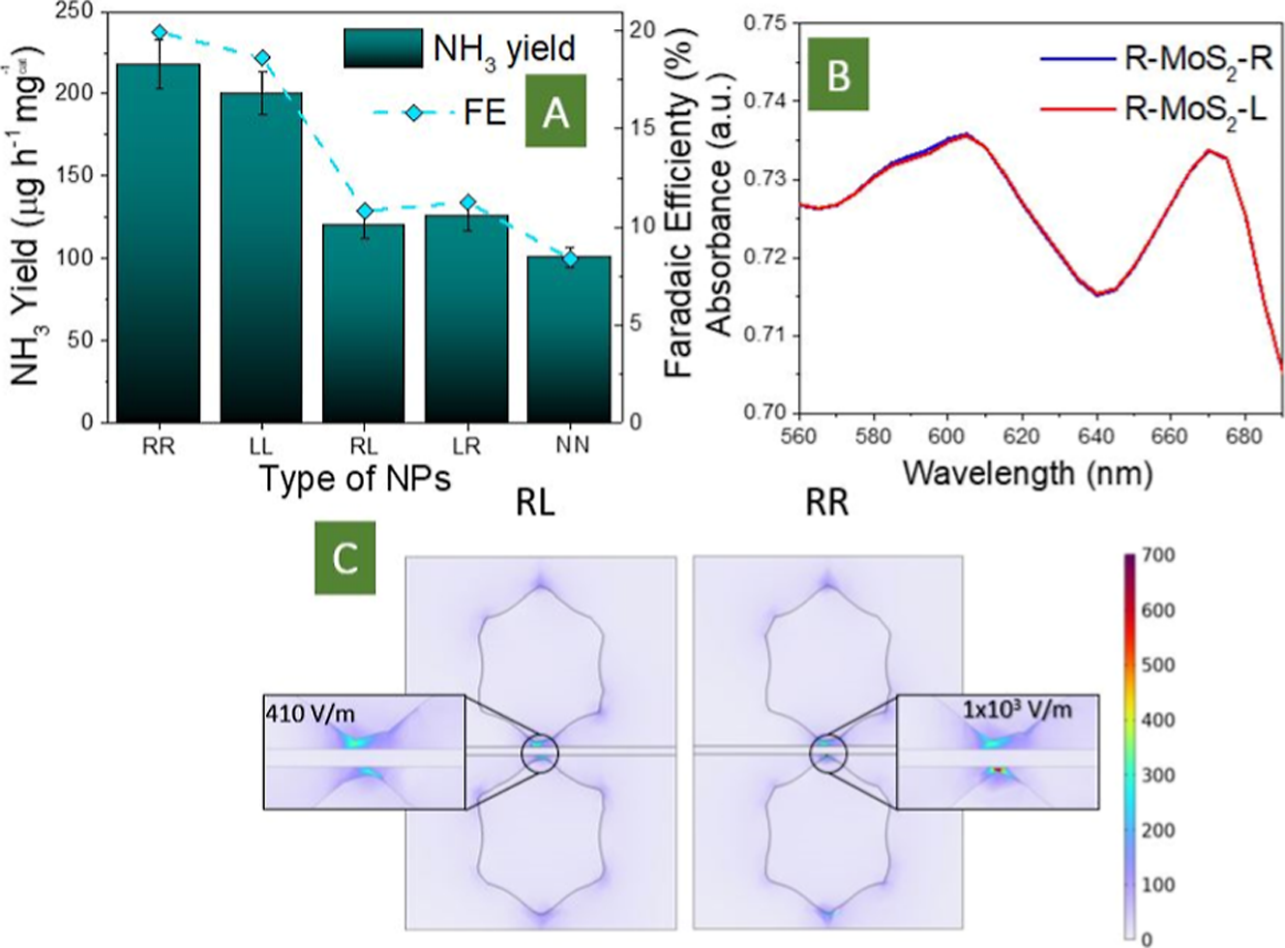
(A) Ammonia yield and Faradaic efficiency obtained with utilization of MoS_2_ flakes sandwiched between chiral (or nonchiral) metal nanoparticles; (B) simulated absorption spectrum for R-MoS_2_-R and R-MoS_2_-L cases; (C) calculated plots of EF intensity and its maximal values (near MoS_2_ surface), reached due to chiral-dependent coupling of Au helicoids. The cut plane was specified to cut diagonally through the particles.

Finally, we also estimated the impact of the value of chirality of AuNPs, used for sandwiching of MoS_2_ and triggering of its catalytic activity in the proposed chiral lock system. For this goal, we used the previously prepared Au nanoparticles with a lower value of chirality (Figures S17, S18 and S35). Like in the previously described case of the sandwiched PS layer, we also observed the apparent dependency of the ammonia production yield on the values of Au helicoid chirality (Figure S36). In particular, the highest rate of NRR was observed even for more chiral nanoparticles. The decrease in nanoparticles chirality apparently results in the decrease of ammonia produced. For the least chiral nanoparticles, we obtained a value of the HPP efficiency close to that of the nonchiral cubes (Figure S36 vs Figure 6A).

Hence, the contributions of matched chirality (and related increase of local EF value in plasmonic hot spot) to the plasmonic coupling are proven to have a significant impact on the triggering of the catalytic activity of MoS_2_ (compared to both mismatched chirality and nonchiral nanoparticles cases), and therefore the utilization of plasmon-active diastereomers makes sense.

To additionally confirm this assumption, we performed a range of additional electromagnetic simulations of plasmon-related EF distribution using COMSOL. The simulated UV–vis absorption of the simulated sandwiched structure is presented in Figure 6B for two combinations, R–R and R–L, where the first letter represents the particle at the sample top. The shape of the curves is similar to the measured absorption spectra shown in Figure 3F. The appearance of two characteristic plasmon absorption bands is replicated by the simulation results and corresponds to different modes. The simulated EF intensities (norm *E* values) at 580 nm excitations are plotted in a vertical cross section of the structure in Figure 6C. The values in the top left and right corners are the maximum EF intensity found in the plots. The appearance of coupled plasmon-related hot spots is well evident in this case (light illumination under an angle is applied, in this case to match the experimental results). Furthermore, difference in plasmon coupling can be attributed to the difference in maximum intensities between the R–R and R–L combinations. We also performed the integration of plasmon-related EF under illumination. The obtained values, highlighted in Figure 6C, were 410 V/m for the R–L combination and close to 1000 V/m for the R–R combination. Hence, the simulation results reveal that the right combination of nanoparticles chirality, i.e., its coincidence, leads to a more efficient plasmon coupling and more efficient light energy concentration in the space of MoS_2_. The results of numerical simulation explain the present experimental results on the efficient triggering of sandwiched MoS_2_ flakes by plasmonic diastereomers.

## Conclusion

The plasmon active chiral diastereomer (called a chiral plasmonic lock) was employed for the triggering of the catalytic activity of 2D MoS_2_ flakes. The flakes were sandwiched between an array of chiral nanoparticles (gold helicoids) with similar or opposite chirality. Due to the 2D nature of MoS_2_ flakes, the plasmon coupling occurs near the boundaries of the nanoparticles that are closest to each other. The coupling was a function of mutual nanoparticles’ chirality and the ability of chiral-based plasmonic “light lock” creation (with the corresponding increase of the local EF value), first demonstrated using SERS measurements. In particular, a more pronounced SERS of MoS_2_ was observed in the case of the same chirality of the top and bottom nanoparticles layers. The created system was subsequently used in the photoelectrochemical nitrogen reduction and ammonia production, which proceeds on the MoS_2_ surface. The apparent increase of catalytic activity was observed in terms of ammonia yield and Faradaic efficiency for the case of the same nanoparticles’ chiralities, compared to opposite ones. Moreover, the obtained results over perform the control case of nonchiral nanoparticle utilization (also used in the coupled regime), highlighting the additional contribution of nanoparticle chirality to plasmon coupling and related plasmon activation of MoS_2_ flakes as well. The obtained experimental results were subsequently verified using numerical simulation, which also demonstrated the ability of very efficient coupling of chiral plasmonic nanoparticles separated by the MoS_2_ spacer, with the corresponding increase in the local EF value, which is further responsible for the triggering of structure redox activity. Moreover, the numerical simulation also shows the dependency of the local enhancement of the plasmon-related EF on the chirality of two Au helicoids. Thus, our work demonstrates that the plasmon-related chirality can also be introduced in the common materials and structures design aimed at realization of plasmon-assisted relevant chemical transformation.

## Experimental Section

### Materials

Hexadecyltrimethylammonium bromide (CTAB, 99.0%), l-ascorbic acid (AA, 99.0%), d-cysteine (99.0%) l-cysteine (98.5%), tetrachloroauric­(III) trihydrate (99.9%), deionized (DI) water, ammonium chloride, sodium sulfate (ACS reagent, ≥99.0%), thioacetamide (ACS reagent, ≥99.0%), sodium molybdate dihydrate (≥98.0%), *N*-methyl-2-pyrrolidone (anhydrous, 99.5%), hydrazine hydrate (50–60%), 4-(dimethylamino)­benzaldehyde (98%), hydrochloric acid (ACS reagent, 37,0%), and ammonium photometric kit test (0.010–3.00 mg·L^–1^) (NH_4_^+^) were purchased from Sigma-Aldrich. The Nafion 117 membrane was purchased from Ion-Power. Potassium hydroxide was purchased from Penta s.r.o. (Czech Republic).

### Sample Preparation

#### Synthesis of Chiral Nanoparticles

First, cubic seeds were prepared following a previously reported technique and then the synthesis of chiral nanoparticles was performed according to the published procedure with few modifications.^36,44,45^ In the next step, a growth solution was prepared: 0.8 mL of 100 mM CTAB and 0.2 mL of 10 mM gold chloride trihydrate were added into 3.95 mL of DI water. Then, 0.475 mL of 100 mM AA solution was added into the growth solution to reduce Au^3+^ to Au^+^. Further 0.05 mL of cubic seeds and 0.005 mL of 0.1 mM l- or d- cysteine were added to the growth solution. The growth solution was left for 1 h at 30 °C; during this time, the solution changed color from pink to blue. The solution was centrifuged twice (4000 rpm, 10 min) and dispersed in a 1 mM CTAB solution for further use. Alternatively, for SEM or TEM characterization, the nanoparticle suspension was washed 3 times in sedimentation/redispersion cycles (in this case, the suspension of Au helicoids tends to agglomerate and should be immediately deposited on SEM or TEM grids before losing stability).

#### Preparation of less Chiral Au Nanoparticles

Less chiral Au nanoparticles were prepared using the same procedure, but the growth solution was left (by rapid synthesis stopping using nanoparticles sedimentation under centrifugation) for 15 and 30 min at 30 °C instead of standard 1 h.

#### Synthesis of 2D MoS_2_

MoS_2_ powder was prepared following a previously reported procedure with some modifications.^46^ C_2_H_5_NS and Na_2_MoO_4_ were mixed together in a molar ratio of 1:2 and dissolved in distilled water under vigorous magnetic stirring for 30 min to obtain a homogeneous transparent solution. The obtained solution was then bubbled by nitrogen flowing during 15 min. Then the solution was transferred to a 100 mL Teflon-lined stainless-steel autoclave, heated to 200 °C, and held at the target temperature for 18 h. After natural cooling, the black precipitate was collected by centrifugation, washed several times with DI water and absolute ethanol, and dried under vacuum at 60 °C. Dried MoS_2_ powder was exfoliated using sonication in *N*-methyl-2-pyrrolidone for 3 h. Subsequently, the dispersion was centrifuged for 5 min at 3000 rpm. The supernatant was collected and then precipitated and transferred to ethanol for further utilization.^47^

#### Sample Preparation

Initially, a layer of L- or R-chiral nanoparticles at a concentration of 30 mg mL^–1^ in DI water was added by drop deposition onto titanium foil (conditions were optimized to reach a closely packed monolayer of Au helicoids: slow evaporation at RT in water saturated atmosphere; the substrate was placed strictly horizontally). Once dried, a layer of MoS_2_ was added onto the sample by the spin-coating method (conditions we also optimized to reach a closed to single flakes layer). Briefly, the optimal deposition parameters were found to be a MoS_2_ concentration in ethanol suspension of 0.8 mg mL^–1^, the speed rotation of 400 rpm, and 2 min spin-coating. Finally, the last layer of nanoparticles was drop-deposited (with the same deposition conditions as in the first step). For CD measurements, similar sample preparation (step by step deposition) was performed on optically transparent quartz substrates.

#### Deposition of the PS Layer and Creation of the Sandwiched Structure

The Au–PS–Au structure was prepared by several subsequent steps: (i) first, the layer of Au helicoids was deposited on the Ti surface using drop casting and slow solvent evaporation, leading to spontaneous self-assembly of Au nanoparticles. (ii) In the second step, the layer of PS was deposited on a self-ordered array of Au helicoids using the spin-coating method. The deposition parameters were: 0.1 wt % PS solution (molecular weight of PS was 500 K) in toluene, 1500 rpm, and 500 rmp/sec acceleration. Spin-coating was carried out for 1 min, which was enough to evaporate the solvent and form a thin polymer film. (Note: The thickness of PS was separately adjusted to be 5–10 nm, using separated deposition on silicon and AFM scratch test-based measurements). (iii) After PS deposition, an additional layer of Au helicoids was added using the procedure described above.

### Measurement Techniques

TEM was performed by using a JEOL JEM1010 instrument (JEOL Ltd., Japan) with a SIS MegaView III digital camera. HRTEM measurements were performed using an EFTEM Jeol 2200 FS microscope (Jeol, Japan). The SEM photos were obtained on a LYRA3 GMU (Tescan, CR) microscope with an accelerating voltage of 2 kV. XRD measurements were carried out using the Empyrean, Malvern Panalytical diffractometer with Cu K⟨α⟩ radiation source in 2θ–θ diffraction mode and Bragg–Brentano geometry. Raman and SERS spectra were measured on a DXR3 spectrometer with a 532 nm excitation wavelength (laser power 3 mW). UV–Vis absorption spectra of the nanoparticles were measured using a Lambda 25 spectrometer (PerkinElmer, USA) in the 300–900 nm wavelength range. Alternatively, UV–Vis spectra of the Au helicoids array were measured under the water condition in reflection mode using a HR2000 (Ocean Optics) spectrometer in 400–800 nm wavelength range. CD spectra were obtained using a J-810 spectrometer (Jasco, Japan) with a scanning speed of 100 nm/min, a bandwidth of 1 nm, the standard sensitivity setting, and integration time of 1 s for each spectral point. Measurements were performed using Au helicoid suspension or after the deposition of Au helicoids on microscopic glass.

The *g*-factor was calculated using following equation

$$g=\frac{\Delta A}{A}$$

where Δ*A* is the CD signal and *A* is the corresponding absorbance.

#### Raman Measurements with Different Wavelengths

Raman and SERS spectra were measured on a DXR3 spectrometer with 455, 532, 633, and 785 nm excitation wavelength (laser power was 1, 3, 3, and 3 mW for used wavelengths).

### Electrochemical and Photoelectrochemical NRR

All of the electrochemical measurements were performed using a portable potentiostat PalmSens 4 (Palm Instruments, Netherlands) controlled by the PSTrace 5.9 program. A two-compartment electrolytic cell (H-type, separated by a Nafion membrane) with a three-electrode connection was used. Prior to testing, the Nafion 117 membrane was pretreated with 3.0% H_2_O_2_ and 0.5 M H_2_SO_4_ under 80 °C for 1 h, followed by rinsing with DI water between each step. The samples were used as the working electrodes. An Ag/AgCl (saturated with 3 M KCl) electrode (BVT Technologies, CZ) was used as a reference electrode, and a platinum wire electrode (BASi, USA) was utilized as a counter electrode. All photoelectrochemical measurements were carried out in 0.1 M Na_2_SO_4_, which was purged with different gases (Ar, N_2_) for 30 min before and continuously during the experiment. The measurements were conducted at room temperature under LED illumination (554 nm wavelength) or in the dark mode. A sample surface was illuminated at a 45° angle with respect to the sample surface normal. After the measurements, applied potentials were converted to the RHE potential. All experiments, except the stability tests, were replicated three times, and the calculated values of standard deviation were subsequently used as error bars.

EIS measurements were performed in the frequency range of 0.1–100,000 Hz (applied potential was −1.1 V vs RHE). In the impedance measurements, an equivalent electrical circuit, comprising solution resistance, contact interface resistance, and constant phase element, was used. EIS spectra were measured in the dark or under light illumination.

### Quantification of NH_3_

The quantity of produced ammonia was quantitatively determined by the ammonia photometric kit test (0.01–3.00 mg/L (NH^4+^), Spectroquant, Supelco, Merck) following supplier’s prescribed procedure. The process involved specific steps: 5 mL of the reaction solution was mixed with 0.6 mL of reagent no. 1 (containing sodium hydroxide). Then some amount of reagent #2 was added to the resulting solution, and the mixture was shaken vigorously until the reagent was completely dissolved. After 5 min, reagent no. 3 (containing thymol and 2-propanol) was added to the reaction solution and stirred. The resultant solution was left to stand for 15 min at room temperature and then analyzed by UV–vis absorption spectroscopy. To establish a calibration curve, known concentrations of NH_4_Cl were added to 0.1 M Na_2_SO_4_ and 0.1 M KOH (to introduce the similar values of pH for “real” and calibration solutions) and then analyzed by the method described above. The absorbance value at ∼692 nm was utilized to estimate the yield of ammonia based on the standard curve.

The NH_3_ yields (as a function of catalyst loading) were calculated using the equation

$${NH}_{3}\;yield\;rate=\frac{c_{{NH}_{3}}\times V}{m_{cat}\times t}$$

where *c*_NH_3__ is the total amount of NH_3_ (measured by photometric test), *V* is the volume of the electrolyte, *m*_cat_ is the mass of the catalyst, and *t* is the reaction time.

### Detection of Hydrazine

The yield of N_2_H_4_ was quantified using the hydrazine photometric kit test [0.005–2.00 mg/L (N_2_H_4_), Spectroquant, Supelco, Merck] following supplier’s prescribed procedure. The process involved specific steps: 5 mL of the reaction solution was mixed with 2 mL of reagent #1 (containing 4-dimethylaminobenzaldehyde) to form a yellow compound. The resultant solution was left to stand for 5 min at room temperature and then analyzed by UV–vis absorption spectroscopy. The absorbance value at ∼450 nm was utilized to estimate the yield of hydrazine based on the standard curve.

### Simulation of Helicoids with a MoS_2_ Flake in Between

Numerical simulations were performed using a finite element method-based software, COMSOL v6.0. The simulations were performed by considering an isolated sandwich-like structure with matching and mismatching helicoids. The MoS_2_ flake with its sandwich-like structure was depicted as a thin (5 nm) cuboid in a 3-dimension model, with chiral nanoparticles created above and below (using previously generated STL files). Dielectric properties of MoS_2_ were taken from ref 48. For Au, the optical constant values were extrapolated from data by Johnson and Christy (1972) that are available in COMSOL libraries.

Linearly polarized light illuminates the top of the sandwich-like structure under the 30° angle of incidence (simulation layout are presented in Figure S1). In order to simulate a single particle and MoS_2_ structure, the entire cell (1000 × 1000 × 600 nm^3^) was framed by perfectly matched layers, and a tetrahedral mesh was generated.

The simulations used periodic boundary conditions, where the boundary surfaces have triangular meshing. Tetrahedral meshing was used for the domains that build the geometry inside the boundaries with a maximum mesh element size constraint of 11 nm for the domains of the nanoparticles and the flake and 26 nm in the surrounding dielectric. The perfectly matched layers were meshed using a swept mesh.

## Supplementary Material



## Data Availability

The data presented in this study are available at https://zenodo.org/records/12698915.

## Notes

Simulation layout used for the calculation of the plasmonic hot spot; schematic representation of plasmonic dipole excitation, UV–vis absorption spectrum and DLS-measured size distribution of Au seeds, nanocubes, and helicoids, LED emission spectra, TEM image of Au nanocubes and helicoids, SEM image of the as-prepared MoS_2,_ Raman spectra of MoS_2_, measured before and after exfoliation, description of the optimization of samples preparation, AFM measured samples surface morphology, SEM cross-section of the sandwiched structure, CD spectra of Au helicoid thin films, CD and UV–vis spectra of Au helicoids after the synthesis rapid stopping, shape of L-Au helicoids obtained by synthesis stopping or prolongation, EIS spectra, UV–vis absorption spectra and calibration curve, used for the estimation of NH_3_ and N_2_H_4_ production yield, NH_3_ yield and Faradaic efficiency obtained using different samples (including control experiments), wavelength dependence of ammonia yield and dependence of ammonia yield on the irradiance, temperature-dependent experiments, NH_3_ yield as a function of the chirality of Au helicoids, and results of stability tests (ammonia production and samples characterization) (PDF)
