# Supplementary material for: Light Entrapment by Plasmonic Chiral Lock for Enhancement of 2D Flakes Catalytic Activity
Source: ACS Appl Mater Interfaces. 2025 May 21;17(22):32553–65. doi: 10.1021/acsami.5c08060 (PMC12147081; doi:10.1021/acsami.5c08060)
Supplement: Supplementary file 1 [file am5c08060_si_001.pdf]

Supporting Information for

“Light Entrapping by Plasmonic Chiral Lock for Enhancement of 2D Flakes Catalytic Activity” by

Anastasiia Tulupova[a], Denis Zabelin[a], Andrea Tosovska[a], Polina Bainova[a], Mariia Erzina[a], Anna Zabelina[a], Vasilii Burtsev[a], Anastasiia Skvortsova[a], Marie Urbanova[b], Martin Kartau[c], Affar S. Karimullah[c], Vaclav Svorcik[a], Oleksiy Lyutakov[a,*]

[a] *Department of Solid State Engineering, University of Chemistry and Technology, 16628 Prague, Czech Republic.*

[b] *Department of Physics and Measurements, University of Chemistry and Technology, 16628 Prague, Czech Republic.*

[c] *School of Chemistry, Joseph Black Building, University of Glasgow, Glasgow, G12 8QQ,UK*

Corresponding author: lyutakoo@vscht.cz

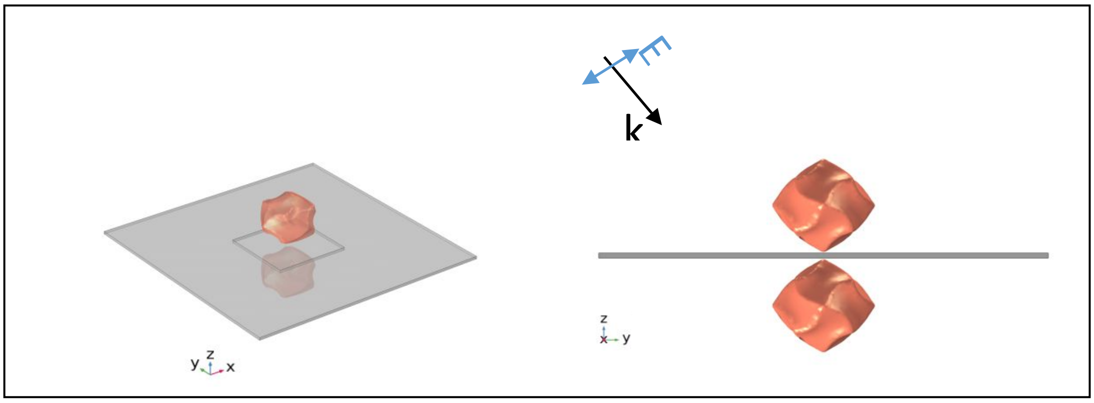

Figure S1 Simulation layout used for the calculation of plasmonic hot spot between coupled Au helicoids with a thin MoS_2 layer between them.

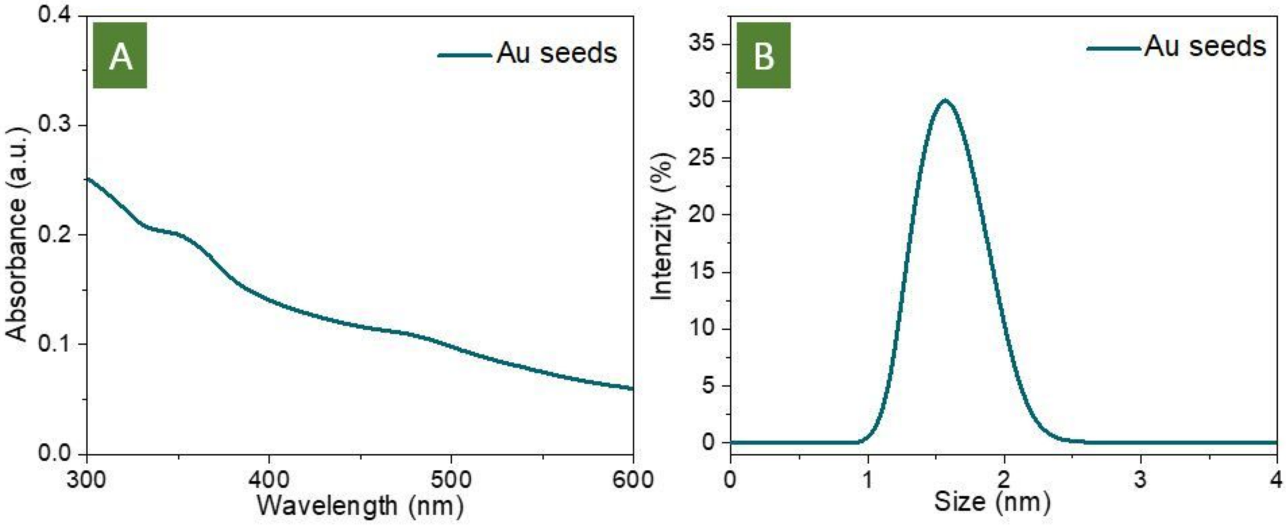

Figure S2 (A, B) - UV-Vis absorption spectrum and DLS-measured size distribution of Au seeds, used for Au nanocubes (and subsequently Au helicoids) synthesis.

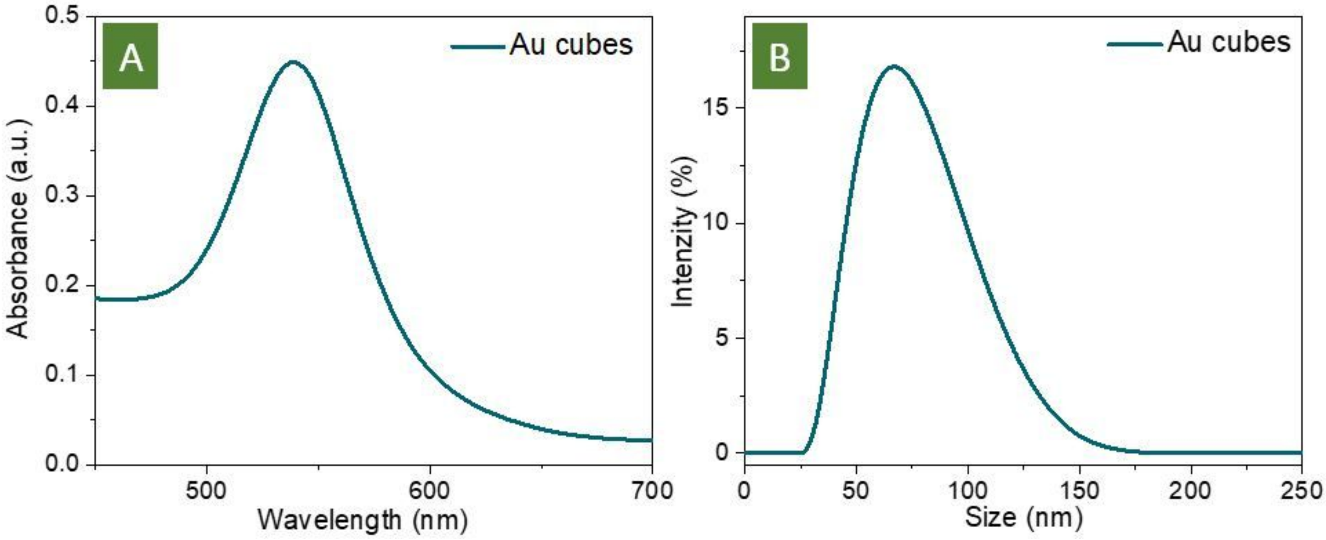

Figure S3 (A, B) UV-Vis absorption spectrum and DLS-measured size distribution of Au nanocubes, used for Au helicoids preparation.

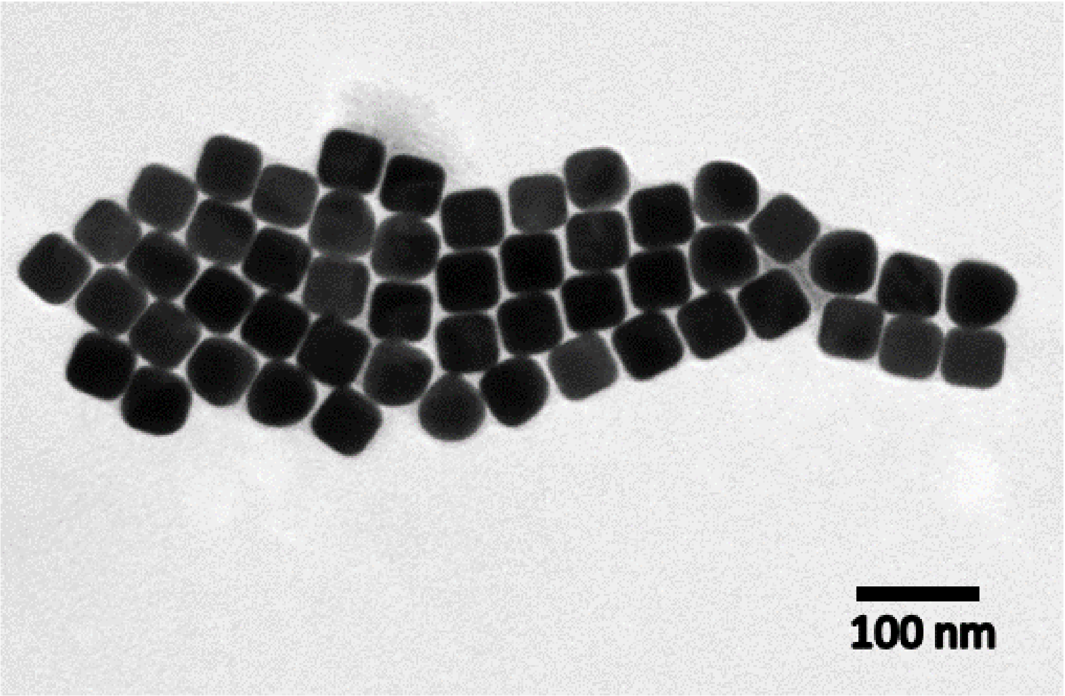

Figure S4 TEM image of Au nanocubes.

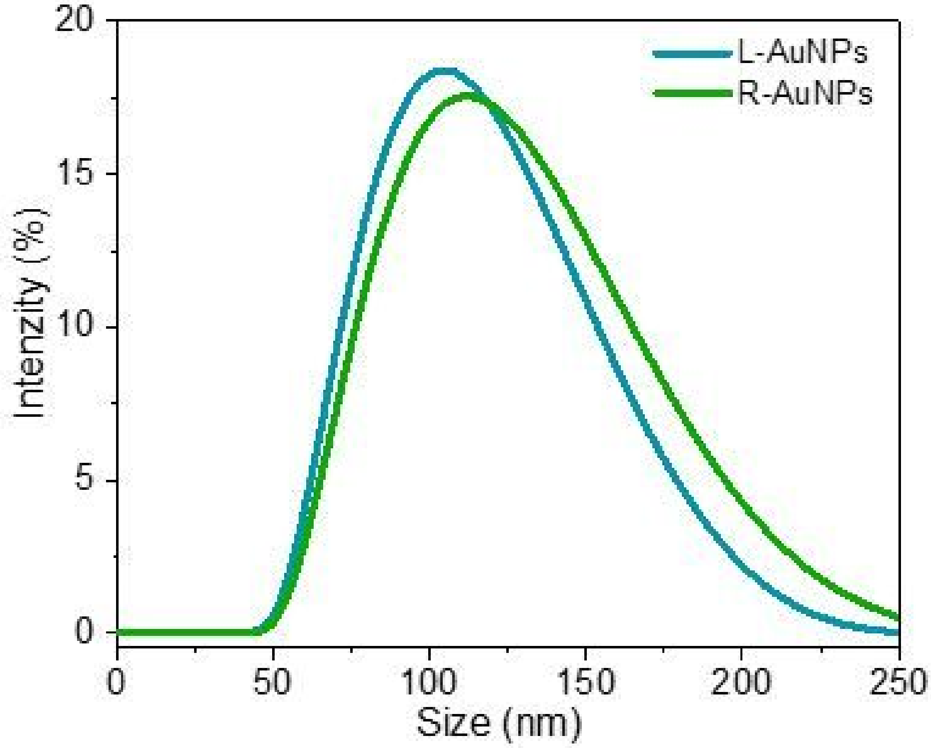

Figure S5 DLS-measured size distribution of Au helicoids.

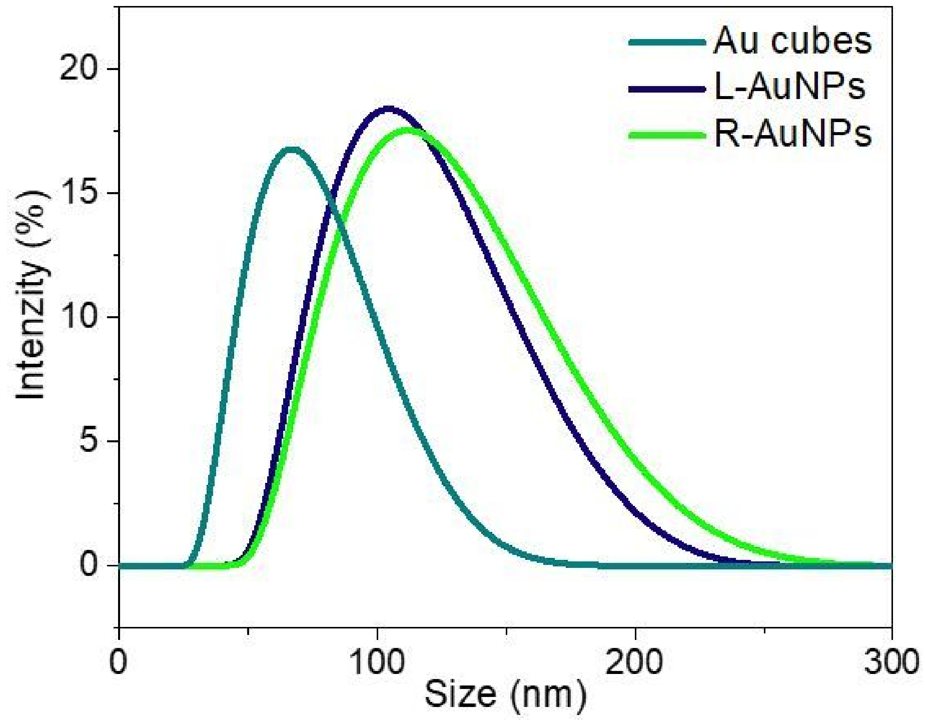

Figure S6 Estimated from DLS size distribution of Au cubes and Au helicoids.

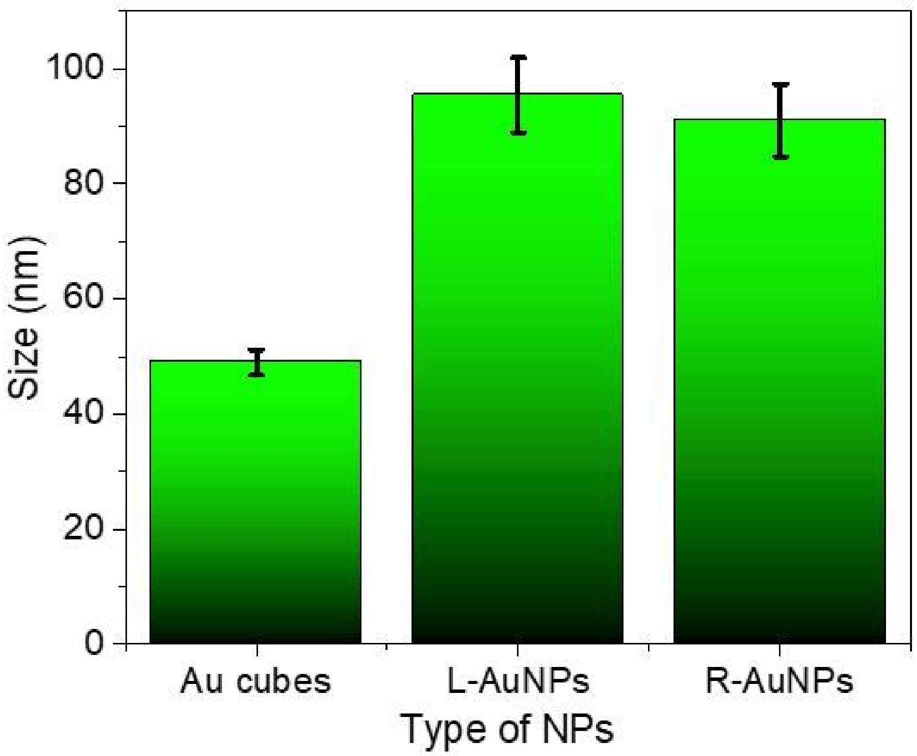

Figure S7 Estimated from SEM and TEM images size of Au cubes and Au helicoids.

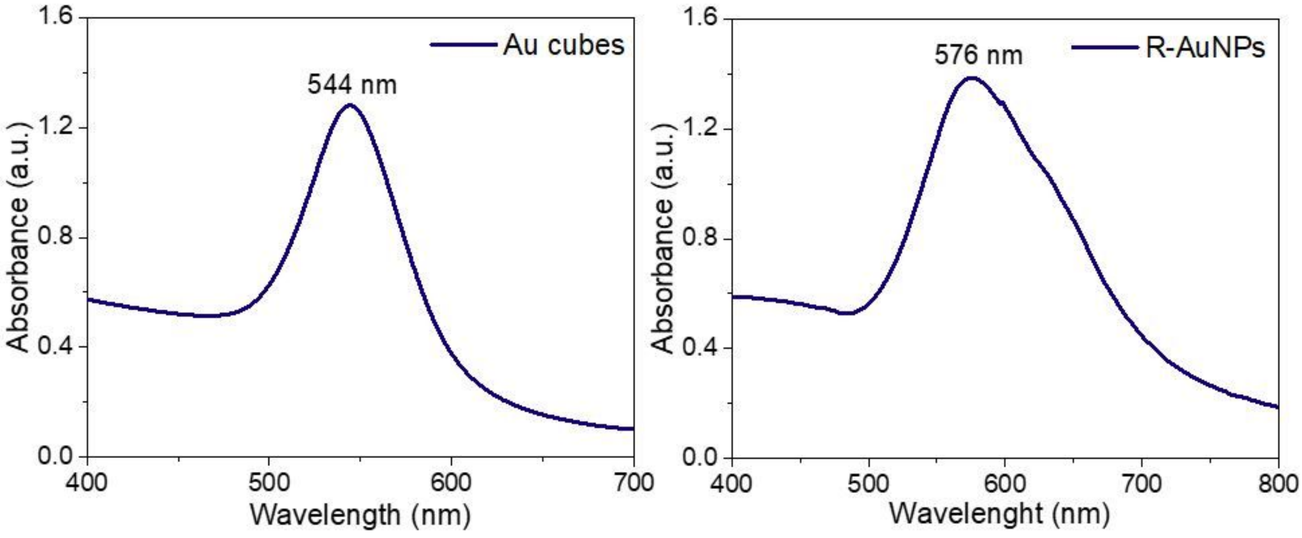

Figure S8 Comparison of UV-Vis absorption spectra of „pristine“ Au nanocubes and subsequently created Au helicoids.

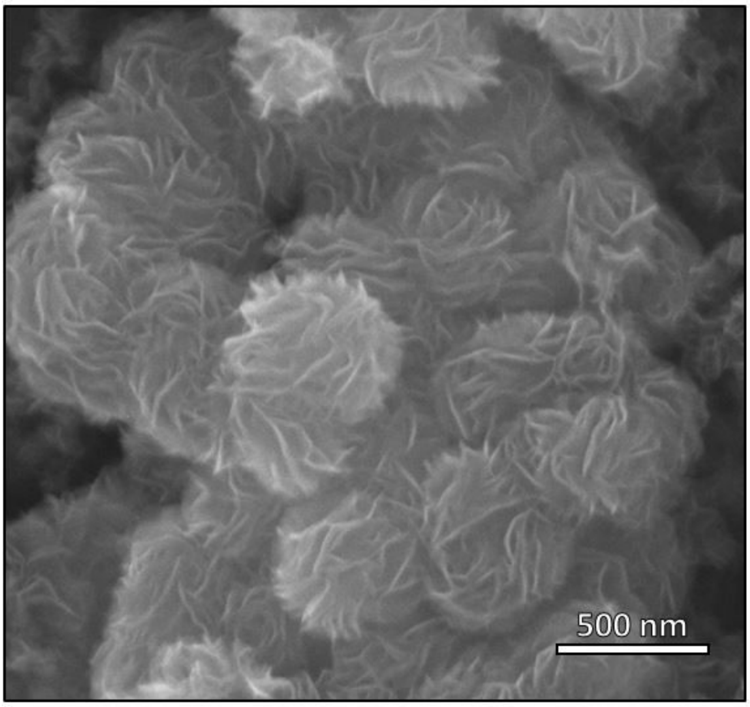

Figure S9 SEM image of as-prepared MoS_2 (before exfoliation).

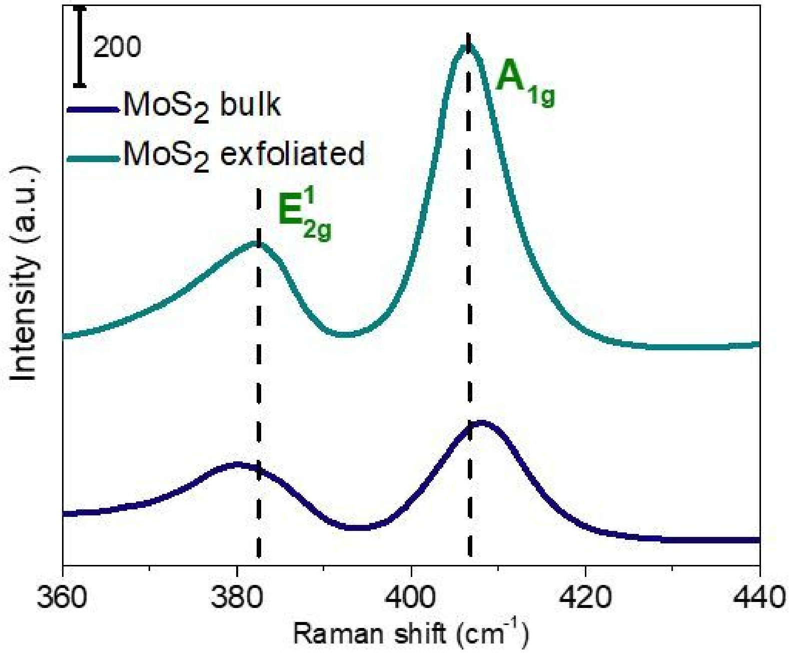

Figure S10 Comparison of Raman spectra of MoS_2, measured before and after exfoliation. After exfoliation the apparent shift of A_{1g} and E^1_{2g} bands was observed, which is in a good agreement with the literature data [S1].

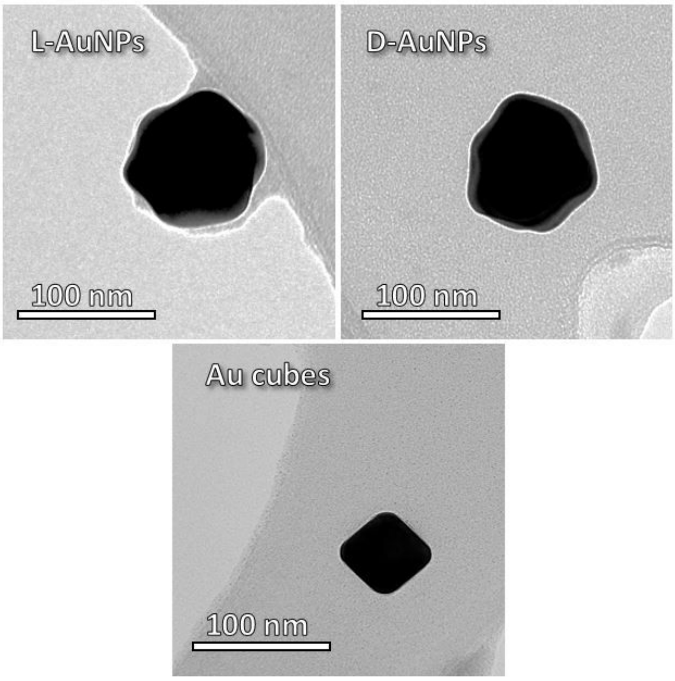

Figure S11 Shape of Au helicoids, depicted in electron transmission mode (TEM). The curved edges of nanoparticles are evident as a grey regions, more transparent for electron beam.

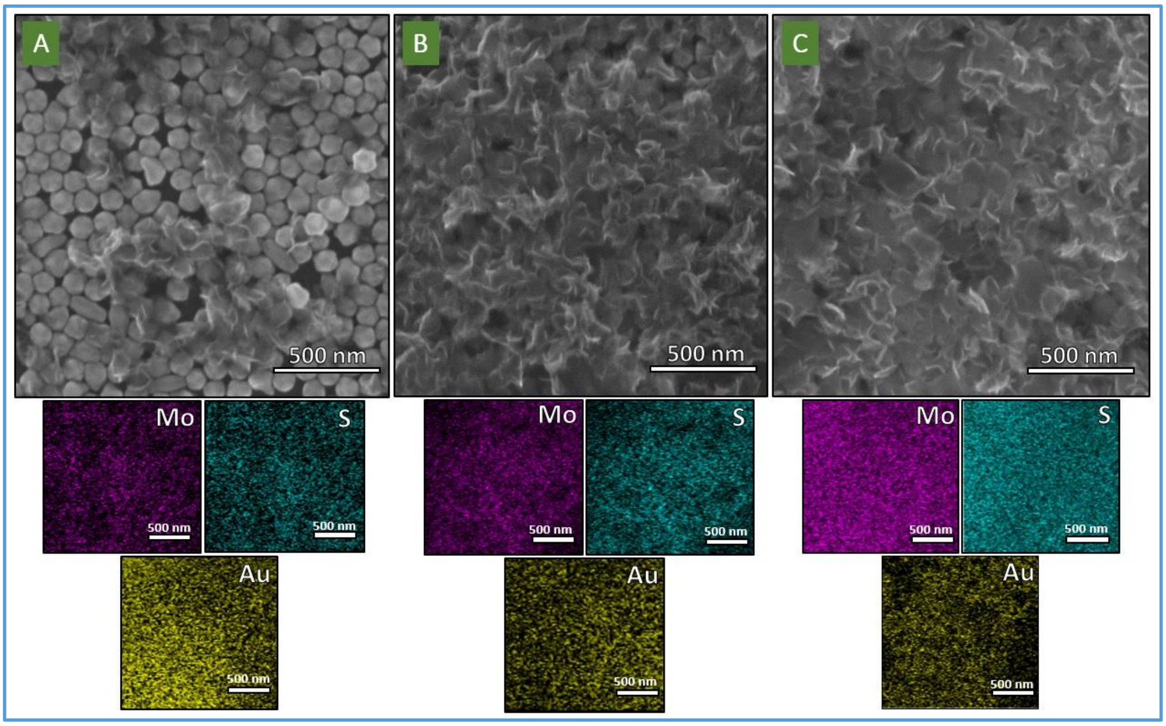

Figure S12 Optimization of samples preparation: coverage of Au helicoids array by MoS_2 flakes: (A) - insufficient coating density (0.4 mg.mL^{-1} solution was used); (B) - an optimal case (0.8 mg.mL^{-1} solution was used), further dilution led to the formation of non-continuous layer, presented in left image; (C) - formation of a too thick MoS_2 layer (1.2 mg.mL^{-1} solution was used, subsequent dilution led to a similar surface morphology and Mo distribution).

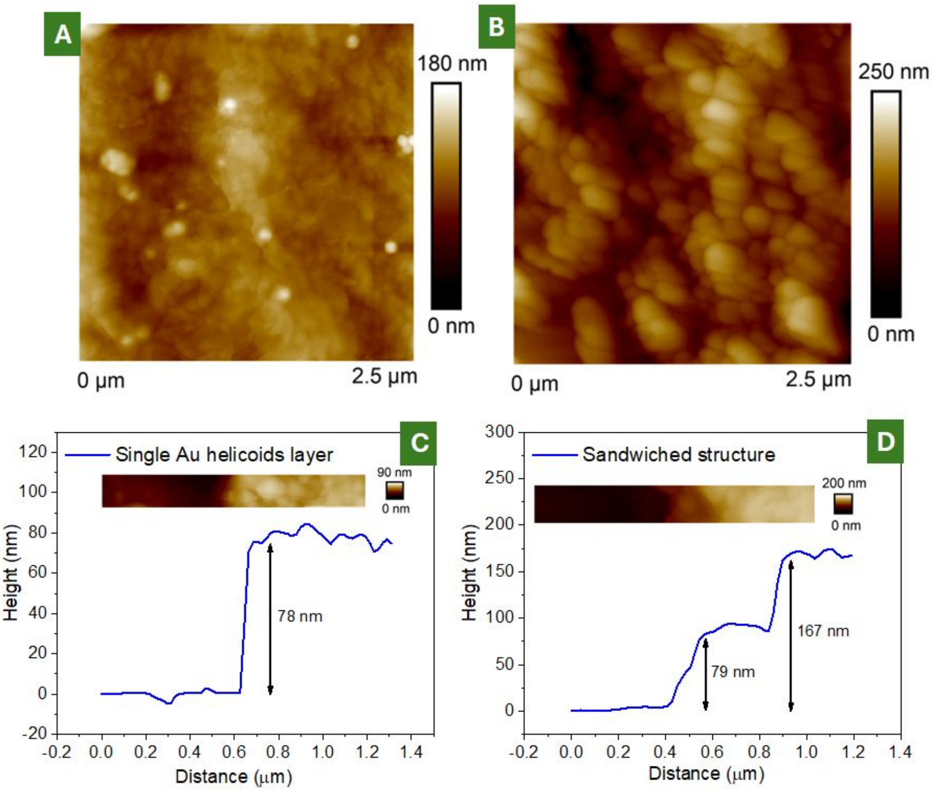

Figure S13 A, B - AFM measured surface morphology of pristine Ti foil and Ti foil with sandwiched R-MoS_2-R structure; C, D – AFM scratch tests results, indicating the thickness of single Au helicoids layer and sandwiched structure.

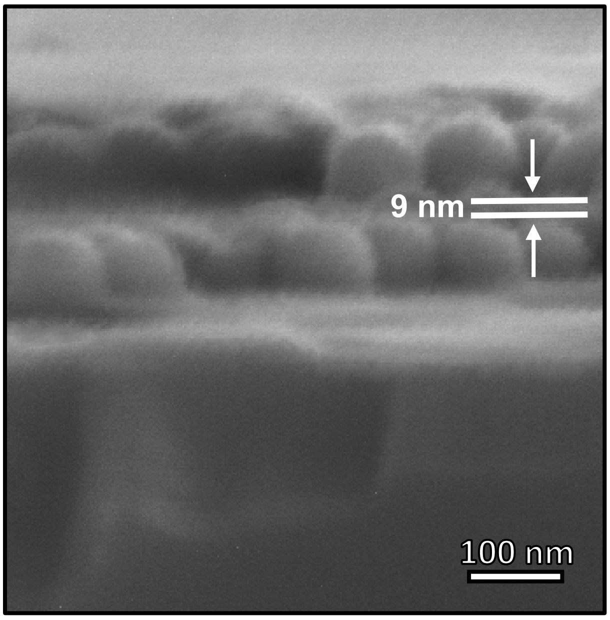

Figure S14 SEM cross-section of sandwiched structure. The thickness of spacer layer was estimated to be near 9 nm.

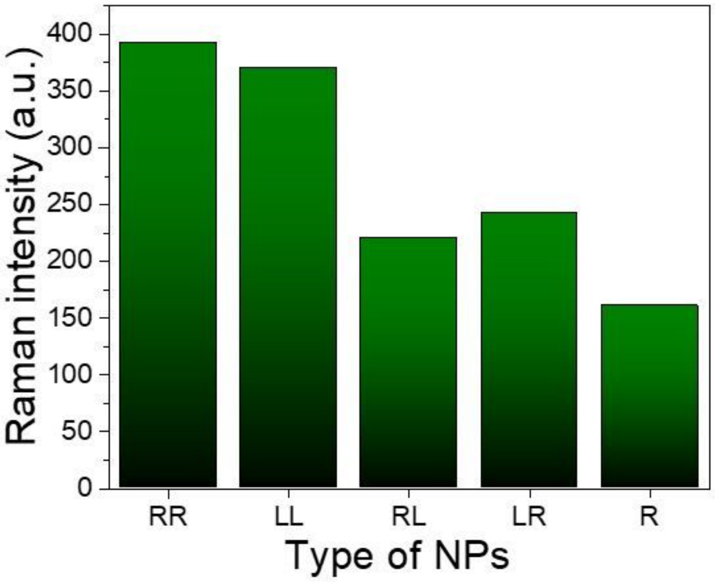

Figure S15 Raman intensity of MoS_2 response as a function of the chirality of Au helicoids.

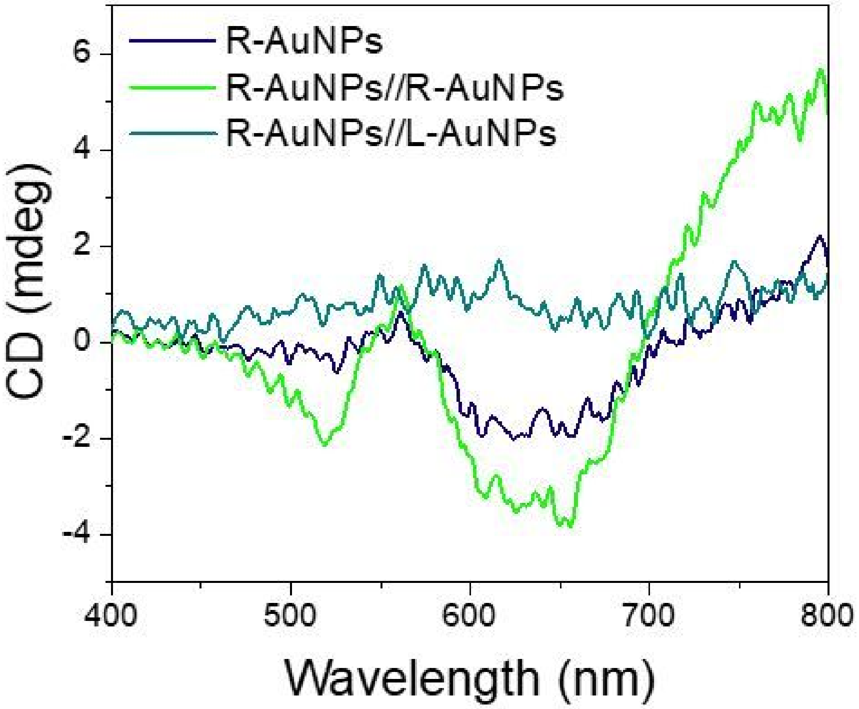

Figure S16 CD spectra of Au helicoids, and sandwiched structures**.**

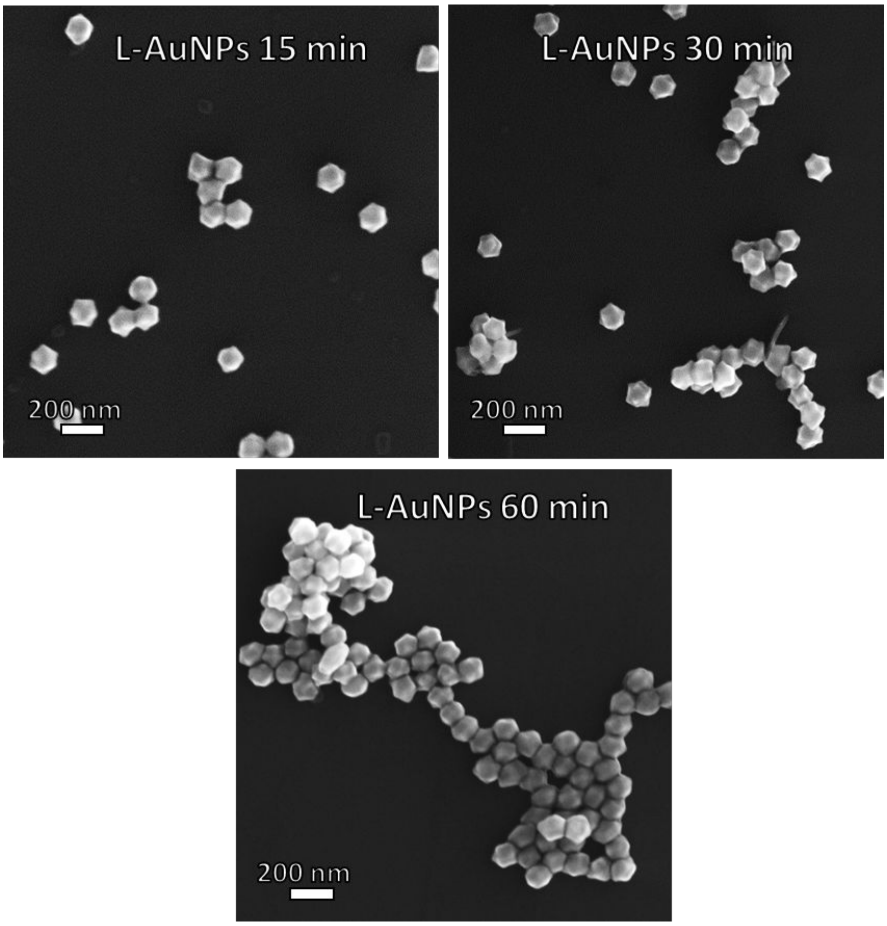

Figure S17 Shape of L-Au helicoids obtained by synthesis stopping after different time intervals.

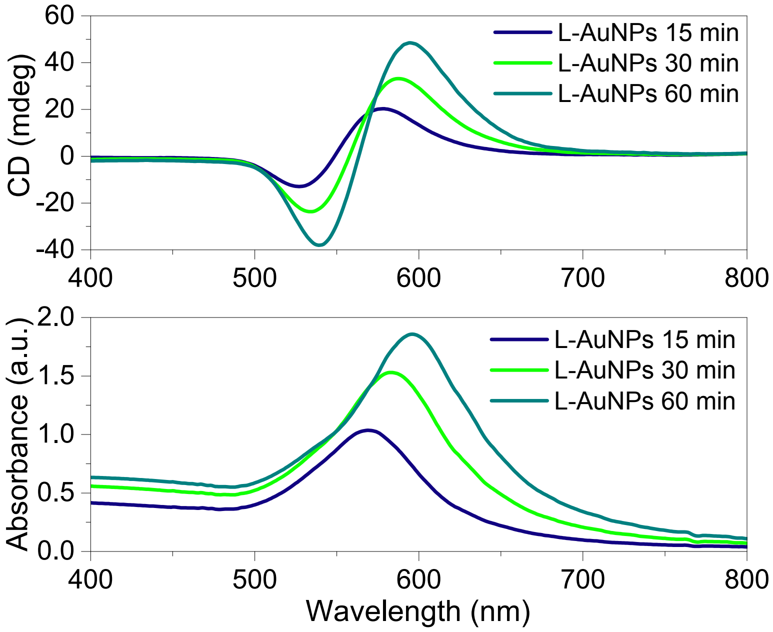

Figure S18 CD and UV-Vis spectra of Au helicoids after the synthesis rapid stopping (15 and 30 min compare to optimal case of 60 min).

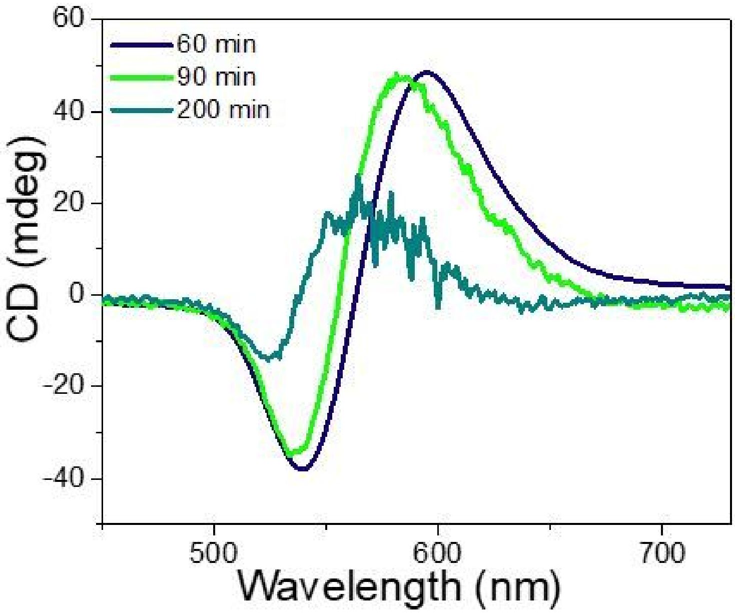

Figure S19 CD spectra of Au helicoids prepared using the prolongation or synthesis time.

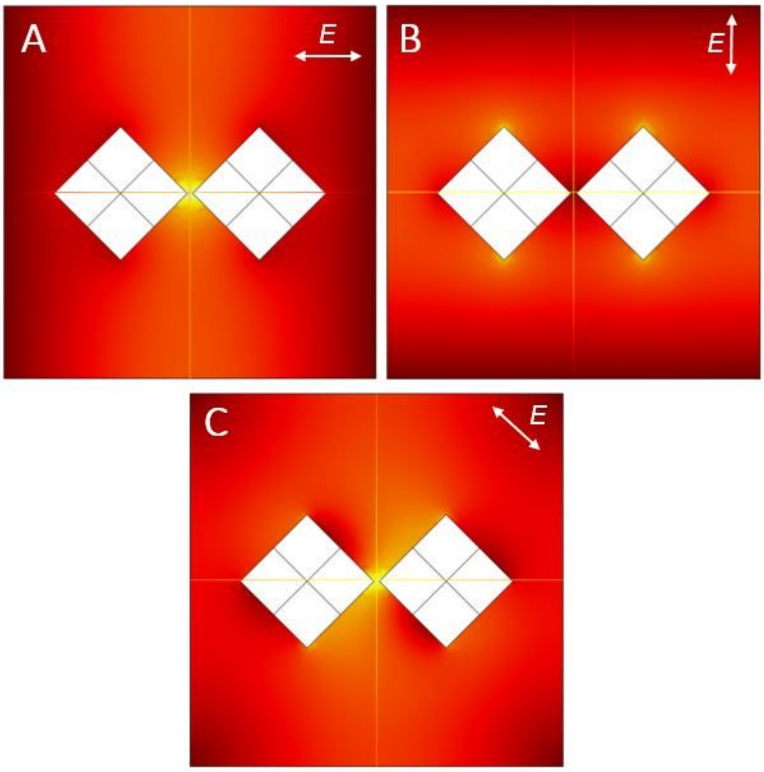

Figure S20 Schematic representation of plasmonic dipole excitation as a function of illumination angle (i.e. electric field orientation) relative to the dipole orientation.

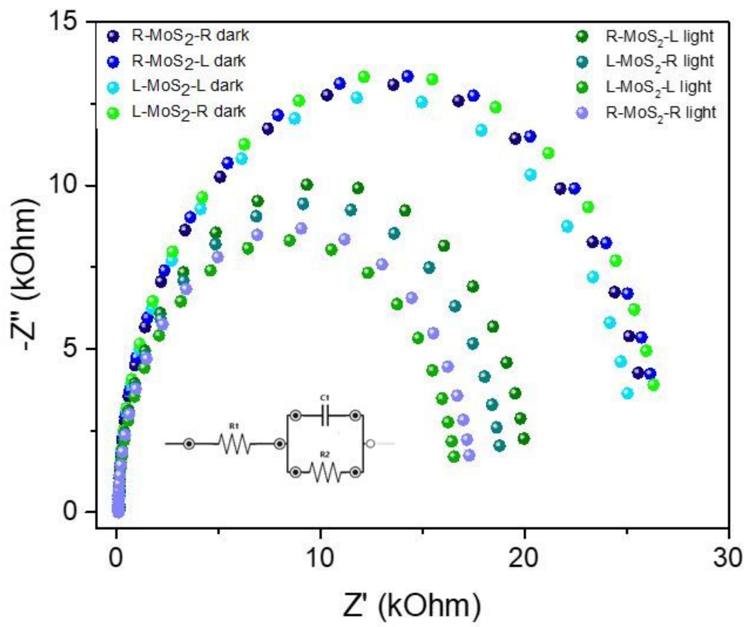

Figure S21 EIS spectra, measured with utilization of MoS2 flakes sandwiched between Au helicoids (coincident or mismatched chirality) with or without light illumination.

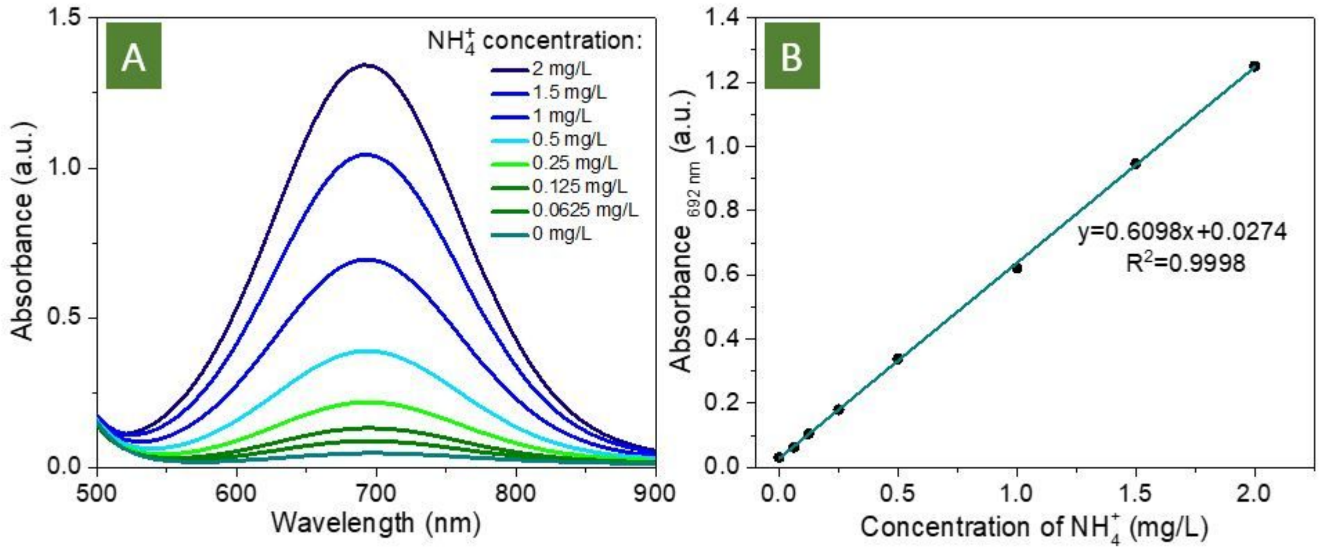

Figure S22 (A, B) - UV-Vis absorption spectra and calibration curve, used for the estimation of NH_3 production yield.

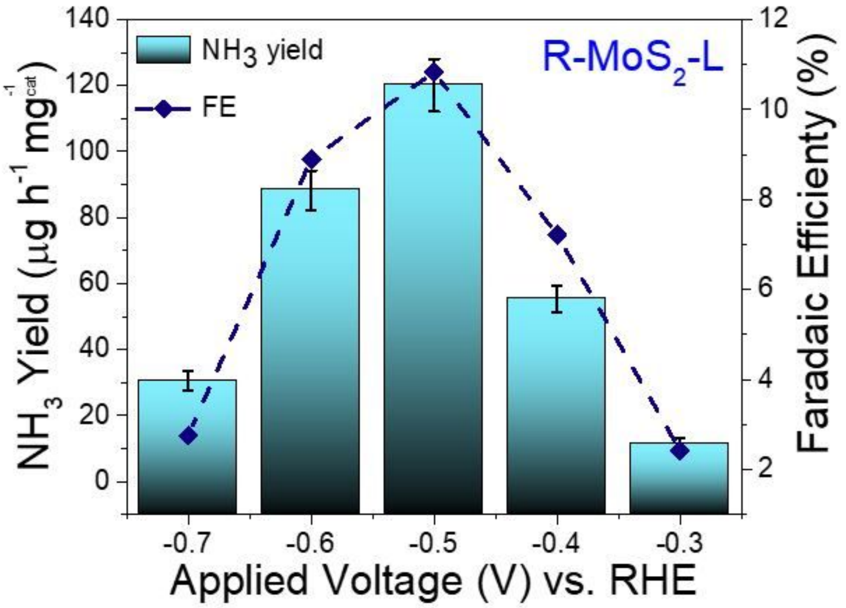

Figure S23 NH_3 yield and Faradaic efficiency obtained with in photoelectrochemical mode with utilization of R-MoS_2-L photoelectrode.

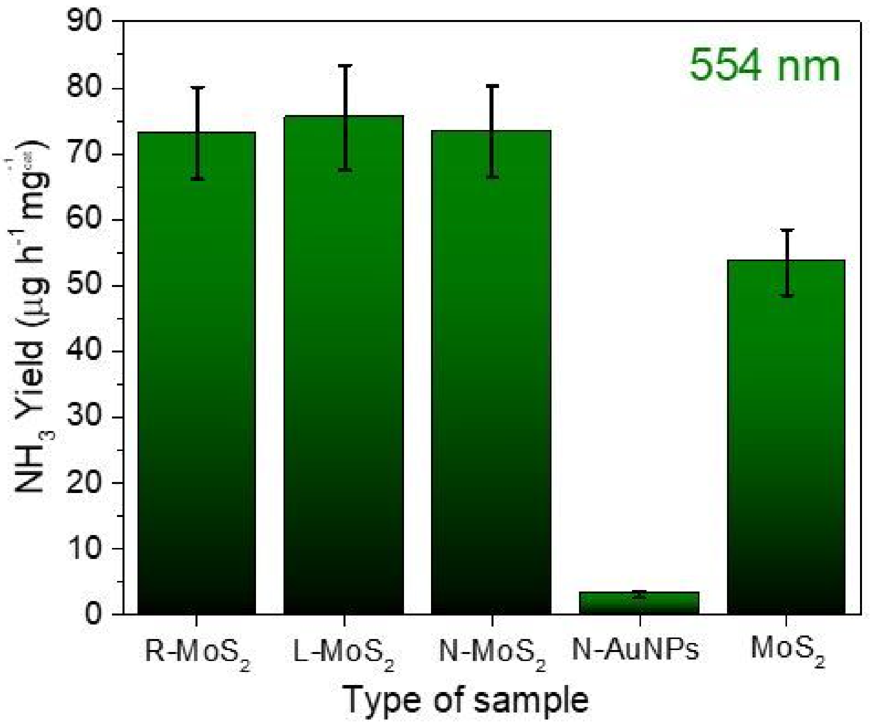

Figure S24 Results of control experiments: NH_3 production yield reached without the sandwiching of MoS_2 flakes between nanoparticles layers or without the addition of MoS_2 on surface of nanoparticles layer.

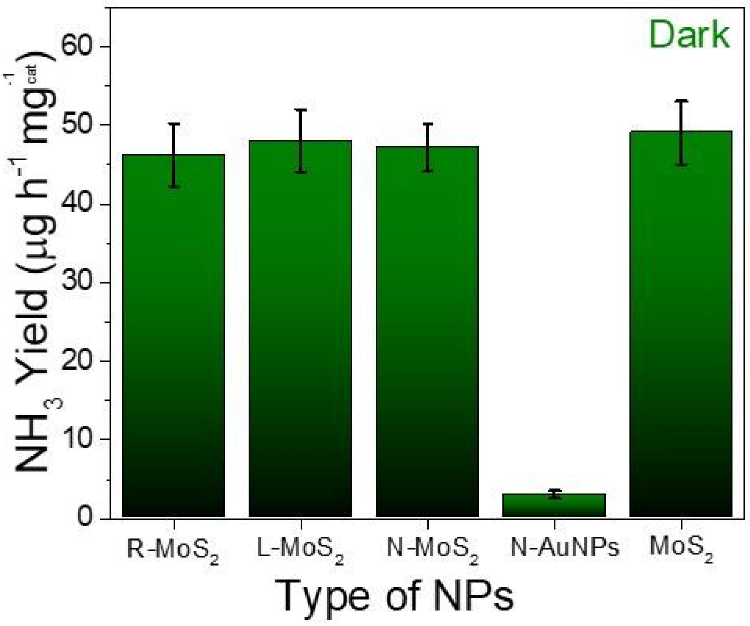

Figure S25 Result of control experiments: NH_3 production yield reached in the dark with utilization of MoS_2 sandwiched between Au nanoparticle layers or directly deposited on the surface of Au nanoparticle layer (N-AuNPs case corresponds to non-chiral Au nanocubes).

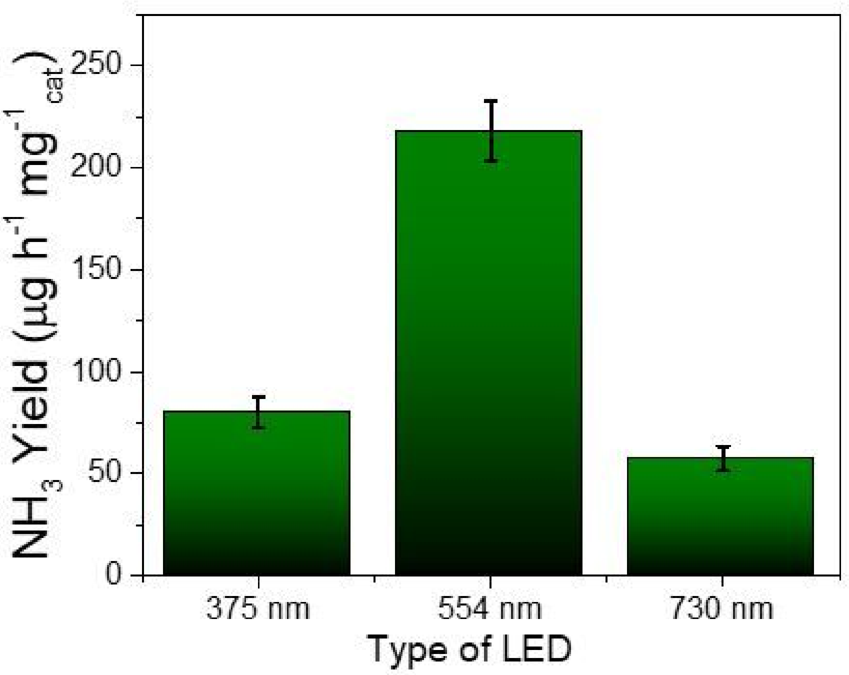

Figure S26 Wavelengths dependence of ammonia yield: comparison between „plasmonic" wavelength (554 nm) and wavelengths not overlapping with plasmonic band.

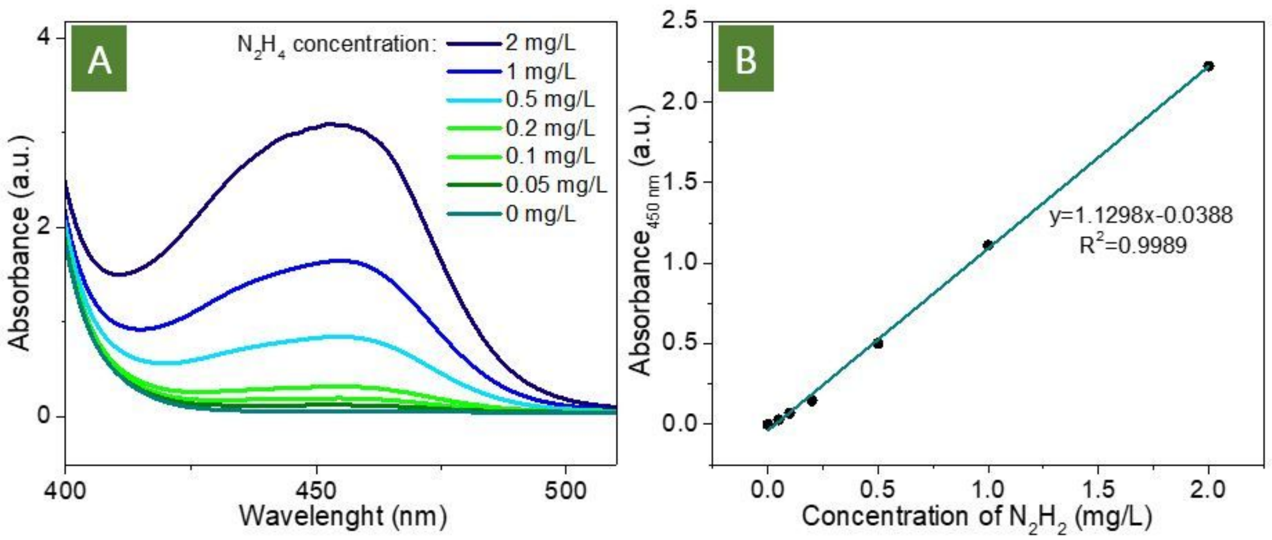

Figure S27 (A, B) **-** UV-Vis absorption spectra and calibration curve, used for the estimation of N_2H_4 production yield (no N_2H_4 was detected in control experiments).

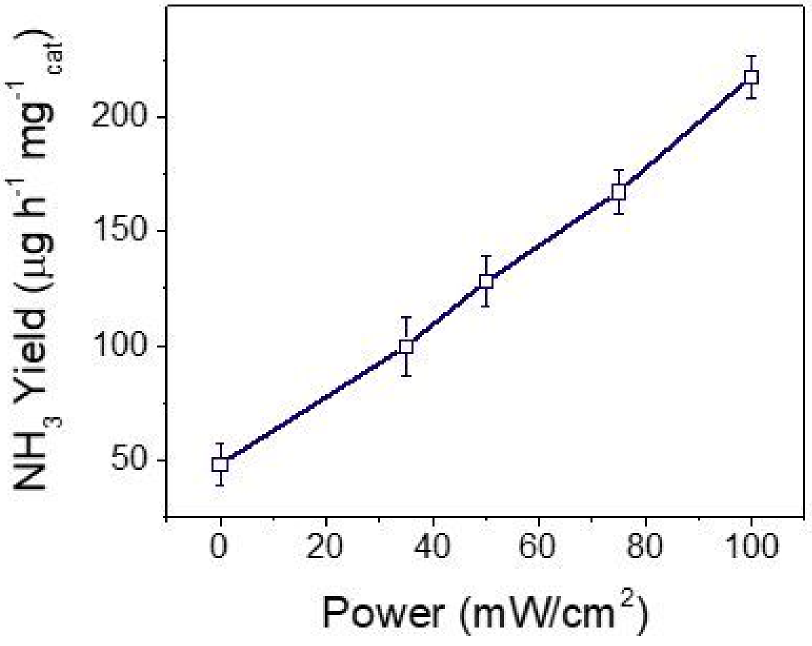

Figure S28 Dependence of ammonia yield on the irradiance (R-MoS_2-R sample).

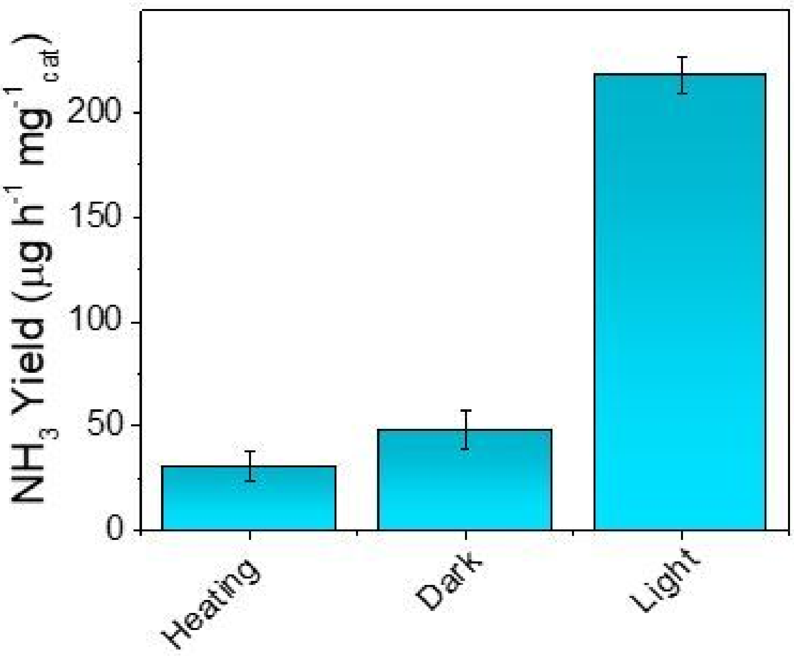

Figure S29 Comparison of ammonia yield, obtained under dark at RT or under increased temperature (60 °C) or under light illumination (R-MoS_2-R sample).

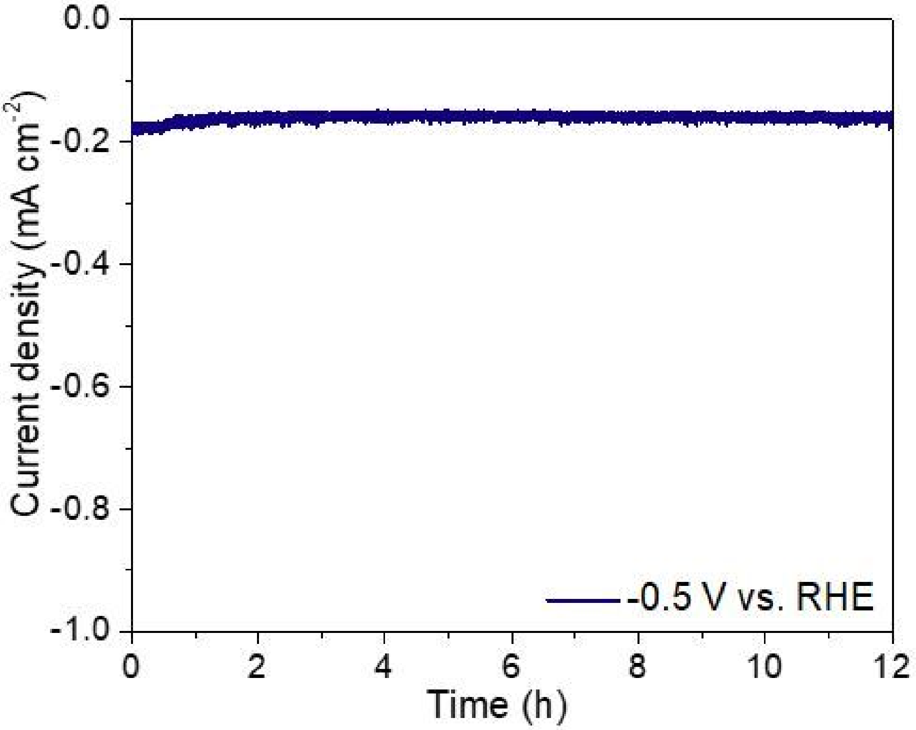

Figure S30 Current density, measured in photoelectrochemical mode with utilization of R-MoS_2-R sample.

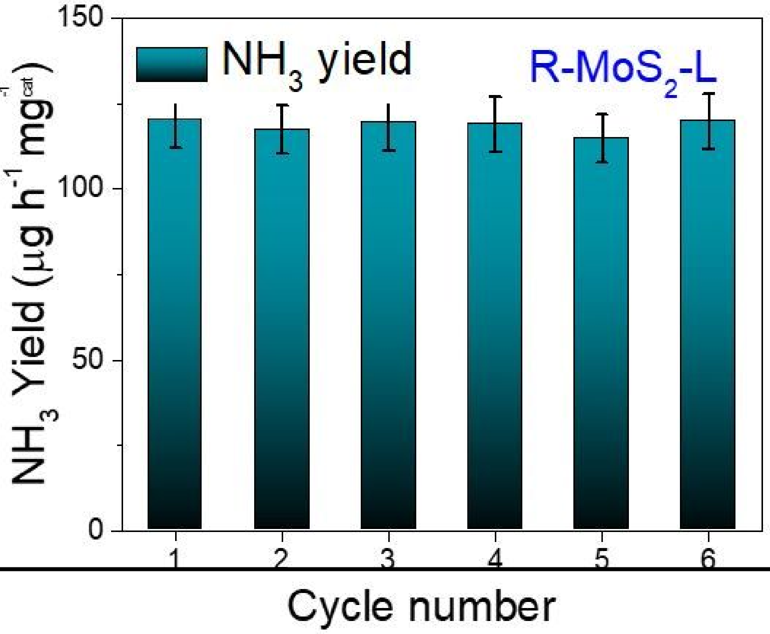

Figure S31 Results of stability tests with each cycle duration of 1h with utilization of R-MoS_2-L photoelectrode.

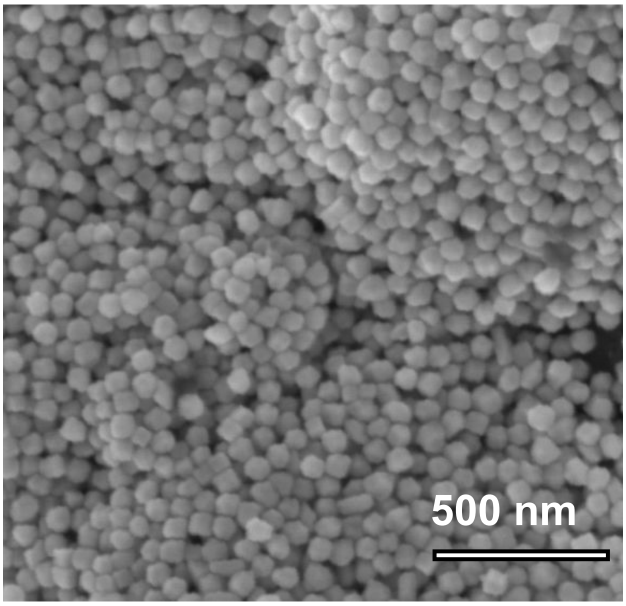

Figure S32 SEM measured surface morphology of R-MoS_2-R sample after the stability test (5 cycles of NH_3 production, with 1h duration of each cycle).

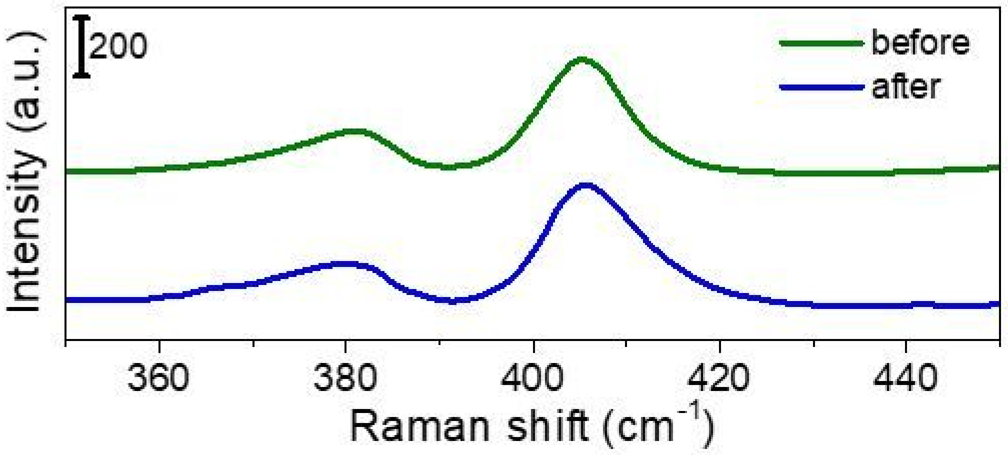

Figure S33 SERS response of sandwiched layer MoS_2 after stability tests 5 cycles, with 1h duration).

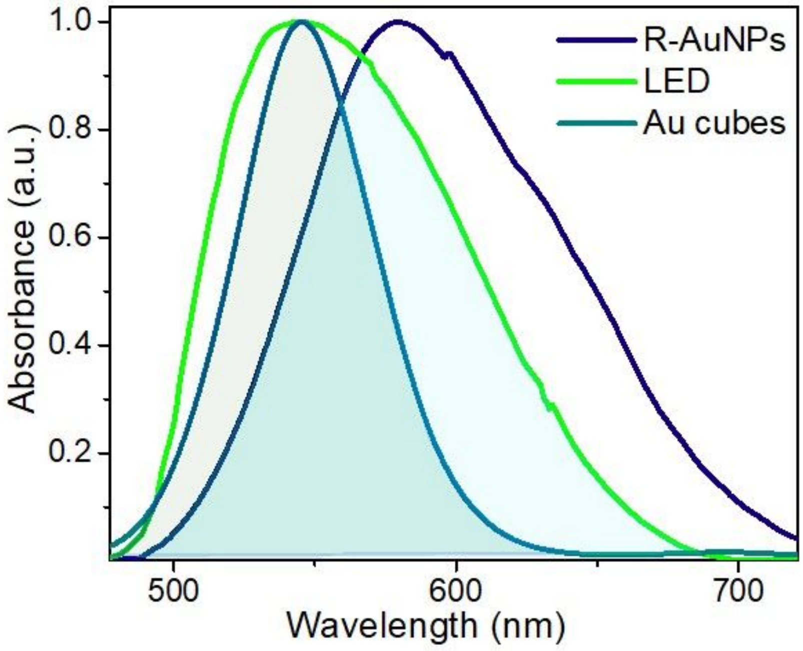

Figure S34 Intersection of LED emission spectrum and plasmon absorption bands of Au helicoids and cubic Au nanoparticles.

Figure S34 – related remark.

The efficiency of plasmonic triggering can be estimated (approximately) as the area of intersection of the normalized absorption spectra (after baseline correction) and the emission spectrum of the LED used (Figure S34 shows the intersection of absorption and emission spectra for Au cubes and Au helicoids). These areas were 77,9 and 60,6 (arbitrary units), the difference between them was closed to 19,9 %, while the efficiency of difference in the NH_3 production was 53,9 %

(main manuscript text, Figure 6A and related discussion). Thus, the observed contribution of chirality can hardly be considered as a simple shift of the absorption bands (due to Au cubes exchange for Au helicoids) with respect to the LED emission spectrum (within the approximation used).

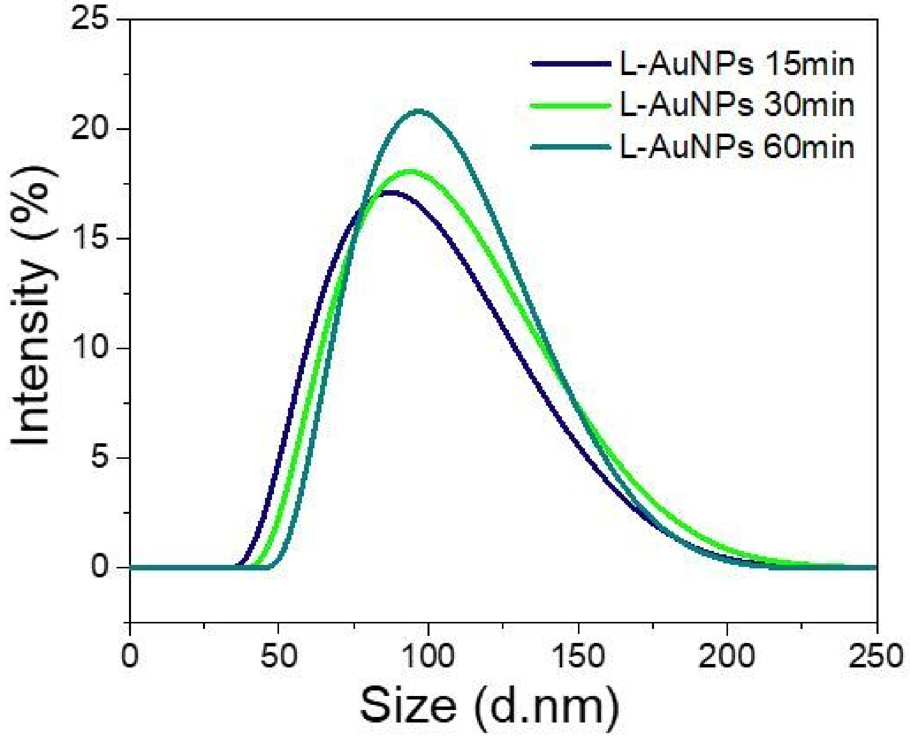

Figure S35 DLS-measured size distribution of Au helicoids prepared by synthesis interrupted after 15, 30 and 60 minutes.

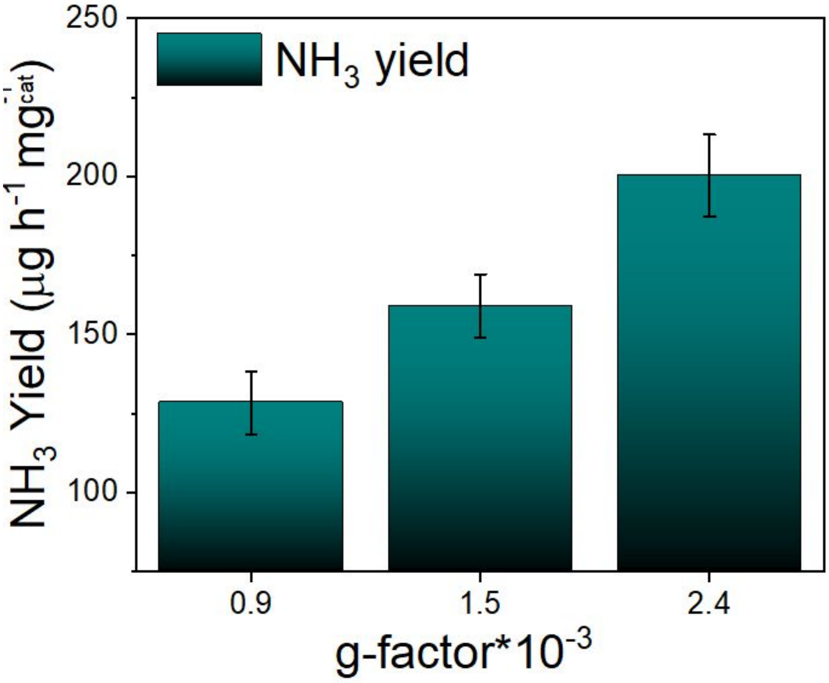

Figure S36 NH_3 yield as a function of the chirality of Au helicoids, used for sandwiching of redox-active MoS_2 and ammonia production in photochemical regime.

References

[S1] Li, H.; Zhang, Q.; Yap, C. C.; Tay, B. K.; Edwin, T. H.; Olivier, A.; Baillargeat, D. From Bulk to Monolayer MoS2: Evolution of Raman Scattering. *Adv. Funct. Mater*. **2012**, 22 (7), 1385-1390, DOI: 10.1002/adfm.201102111
